# Integrative Analysis Identified an Eight-Gene Risk Signature Linked to CDK7 and Explored Its Association with HCC Progression via RelA Phosphorylation

**DOI:** 10.32604/or.2026.081711

**Published:** 2026-07-16

**Authors:** Bin Lan, Jie Tan, Qing Wang, Siyuan Zeng

**Affiliations:** 1 Department of Interventional and Vascular Surgery, Hunan Provincial People’s Hospital (the First Affiliated Hospital of Hunan Normal University), Changsha, China; 2 Department of Breast and Thyroid Surgery, Hunan Provincial People’s Hospital (the First Affiliated Hospital of Hunan Normal University), Changsha, China

**Keywords:** Hepatocellular carcinoma, cyclin-dependent kinase 7 (CDK7), prognostic genes, risk model, hepatocellular carcinoma (HCC) prognostic biomarker

## Abstract

**Backgrounds:** Cyclin-dependent kinase 7 (CDK7) plays key roles in transcription and cell cycle regulation, and its inhibition has been proposed as a potential therapeutic strategy for hepatocellular carcinoma (HCC). The primary research objective of this study is to identify and validate CDK7-associated prognostic genes in HCC using bioinformatics approaches, construct a reliable prognostic risk model, and explore the functional role of CDK7 in HCC progression through *in vitro* and *in vivo* experiments, with a specific focus on its association with RelA/p65 phosphorylation, so as to provide evidence supporting CDK7 as a potential prognostic biomarker and therapeutic target for HCC. **Methods:** Two independent HCC cohorts from The Cancer Genome Atlas (TCGA)-HCC and the Gene Expression Omnibus (dataset GSE14520) were analyzed. Patients were stratified by *CDK7* expression. Differentially expressed genes were identified in both CDK7-based and tumor-versus-normal comparisons, and the intersection of these genes was analyzed. Univariate Cox regression and machine learning were used to screen prognostic genes and construct a risk model. The model’s predictive utility was assessed using Kaplan–Meier and receiver operating characteristic (ROC) analyses. Mutation landscapes were compared between risk groups. The functional association of CDK7 was validated through *in vitro* and *in vivo* experiments. **Results:** Eight prognostic genes (*CHGA*, *SH2D5*, *ACTBP12*, *SOX2*, *AGR2*, *ISM2*, *KRT12*, and *BRDT*) were screened from 98 intersecting genes via univariate Cox regression and least absolute shrinkage and selection operator regression analyses to construct a risk model. In the TCGA-HCC (374 HCC samples and 50 control samples) and GSE14520 cohorts (222 HCC samples and 212 control samples), patients in the high-risk group had significantly worse overall survival than those in the low-risk group (Hazard Ratio [HR] = 0.003–0.036, 95% Confidence Interval [CI] = 1.01–4.28, *p* < 0.05). The predictive accuracy of the model was moderate, with the areas under the ROC curves (AUCs) for 1-, 3-, and 5-year survival exceeding 0.6 (specifically, 0.69 for 3-year survival). *In vitro* and *in vivo* experiments demonstrated that *CDK7* expression is associated with HCC proliferation, migration, invasion, and tumor growth, and this association might be related to RelA/p65 phosphorylation. **Conclusions:** This study identified a novel eight-gene prognostic signature linked to CDK7 in HCC and provided experimental evidence that CDK7 promotes tumor aggressiveness. These findings suggest the potential of CDK7 as a prognostic biomarker and therapeutic target for HCC, though further validation of its clinical utility is needed.

## 1 Introduction

Hepatocellular carcinoma (HCC) is recognized as the most common primary malignancy of the liver and is a major public health concern globally [1]. HCC is the sixth most common cancer globally and the fourth leading cause of cancer-related mortality, causing more than 800,000 new cases and approximately 700,000 deaths annually [2]. The incidence of HCC is particularly high in East Asia and sub-Saharan Africa, where it is often linked to chronic liver diseases, particularly cirrhosis. This condition is frequently caused by persistent infections by the hepatitis B virus (HBV) or hepatitis C virus [1,3]. In addition to these viruses, the risk factors for HCC include nonalcoholic fatty liver disease, excessive alcohol consumption, exposure to hepatotoxins, and certain rare genetic disorders [3,4,5]. In addition, HCC has obvious familial clustering, and genetic factors also play a key role in the risk of HCC development [6]. The consensus indicates that HCC is characterized by a complex interaction of genetic and environmental factors. HCC often presents at an advanced stage, with approximately 40%–50% of patients diagnosed after significant disease progression [6,7]. Treatment options vary widely, ranging from surgical interventions, such as liver resection and transplantation, to locoregional therapies such as transarterial chemoembolization and systemic therapies, including targeted therapies and immunotherapy [6,8]. Currently, surgical resection remains the preferred treatment [8,9]. However, HCC has a high recurrence rate, contributing to poor prognosis. Consequently, it is essential to identify more reliable inhibitors or genes to enhance diagnostic capabilities and develop tailored treatment strategies for individual patients.

Cyclin-dependent kinase 7 (CDK7) is a key member of the cyclin-dependent kinase family, which plays an important role in the regulation of various cellular processes, including the cell cycle and transcription. CDK7 primarily functions as a CDK-activating kinase (CAK), phosphorylating and activating other cyclin-dependent kinases essential for cell cycle progression [10]. Furthermore, CDK7 is a component of the transcription factor TFIIH, which is involved in the transcriptional regulation of RNA polymerase II, thereby linking it to the control of gene expression [11]. Given the critical role of the NF-κB pathway (with RelA/p65 as its core subunit) in HCC proliferation and metastasis [12,13], and the potential of CDK7 to regulate RelA phosphorylation as a transcriptional kinase, whether CDK7 drives HCC progression via RelA/p65 phosphorylation remains an urgent scientific question. Studies have found that elevated CDK7 expression is correlated with advanced tumor stages and unfavorable clinical outcomes in patients with HCC, suggesting that CDK7 could represent a potential prognostic biomarker for this malignancy [14]. CDK7 inhibition has been demonstrated to impair transcription and induce cell cycle arrest, particularly at the G2/M phase, which can reduce tumor growth and increase apoptosis in HCC cells [15]. For example, the selective CDK7 inhibitor THZ1 has exhibited significant antitumor activity in HCC models, effectively reducing cell viability and inducing apoptosis through mechanisms involving the downregulation of oncogenes associated with cell cycle regulation and tumor progression [14,15]. Similarly, other epigenetic regulators such as EZH2 have been validated as therapeutic targets in multiple cancers, with inhibitors (e.g., tazemetostat) advancing to clinical use. CDK7, as a core kinase in transcriptional regulation, may hold analogous targeting potential [16].

The potential of CDK7 as a core component of a multi-gene prognostic model remains unclear; whether it drives HCC progression is unestablished, and the lack of studies integrating CDK7 multi-gene signatures with validation of the related molecular mechanism limits the systematic understanding of CDK7’s role in HCC. To address these gaps, this study aimed to identify and validate CDK7-associated prognostic genes in hepatocellular carcinoma (HCC) using bioinformatics approaches based on The Cancer Genome Atlas (TCGA) and Gene Expression Omnibus (GEO) databases, subsequently constructing and validating a reliable 8-gene prognostic risk model for HCC. Furthermore, we sought to explore the functional role of CDK7 in HCC progression through *in vitro* and *in vivo* experiments, with a specific focus on testing the hypothesis that CDK7 promotes HCC aggressiveness via regulating RelA/p65 phosphorylation. Ultimately, this work provides evidence supporting CDK7 as a potential prognostic biomarker and therapeutic target for HCC. Leveraging relevant data from TCGA and GEO databases, we screened for and identified eight prognostic genes associated with CDK7 using bioinformatics methods, constructed and validated the risk model, and further explored the effect of CDK7 on HCC malignant phenotypes through *in vitro* and *in vivo* experiments focusing on RelA/p65 phosphorylation.

## 2 Materials and Methods

### 2.1 Data Collection

Transcriptomic data, patient survival information, and clinical data were obtained for the TCGA-HCC cohort. In detail, the TCGA-HCC dataset, consisting of 374 HCC and 50 control liver tissue samples, was retrieved from the TCGA database (http://cancergenome.nih.gov/) as the training cohort. Furthermore, the GSE14520 dataset was obtained from the GEO database (http://www.ncbi.nlm.nih.gov/geo/) as the validation cohort. Specifically, GSE14520 included 222 HCC and 212 control tissue samples based on the GPL3921 platform. To address technical differences between the TCGA-LIHC (RNA-seq) and GEO (microarray) datasets, we built a multiplatform analysis framework with TCGA-LIHC (374 tumors/50 normals) as the training set and three GEO datasets (GSE14520) as the validation sets. For preprocessing, RNA-seq data were reverted to raw counts, mapped to gene symbols, and standardized (VST/FPKM/TPM), whereas microarray data had probe IDs converted to gene symbols, with duplicate genes removed to retain the highest expression record. For integration, we rejected ComBat batch correction, instead using median grouping (e.g., high/low *CDK7* expression) to build a risk model in TCGA and validate it in GEO. By setting strict thresholds [|log_2_ fold change (log_2_FC)| > 1, false discovery rate (FDR) < 0.05] and a random seed [set. seed (123)], we ensured the analysis was robust, transparent, and reproducible.

### 2.2 Gene Expression of CDK7

The CIBERSORT algorithm in the immunedeconv software package evaluates the infiltration of different immune cell types in high-and low-expression groups. A stacked abundance plot was generated to demonstrate the infiltration status of 22 immune cells. To ascertain whether CDK7 could distinguish between HCC and control samples in the TCGA-HCC cohort, the Wilcoxon test was applied to analyze the differential expression of CDK7 (*p* < 0.05). Subsequently, patients with HCC in TCGA-HCC were divided into high- and low-expression groups based on the median CDK7 expression level. The log-rank test was employed to generate Kaplan–Meier (KM) to explore the expression discrepancy of CDK7 between the two groups (*p* < 0.05, unadjusted).

### 2.3 Function Enrichment Analysis

Gene Set Enrichment Analysis (GSEA) was performed using the “clusterProfiler” (v 4.6.0) package to investigate the biological processes and signaling pathways associated with CDK7, based on the high- and low-expression groups in TCGA-HCC [17]. Significance criteria of |normalized enrichment score (NES)| > 1 and adj. q < 0.25 were applied. The background gene set was “c2.cp.kegg.v7.4.symbols.gmt” sourced from the Molecular Signatures Database (https://www.gsea-msigdb.org/gsea/msigdb/index.jsp).

### 2.4 Immune Infiltration Analysis

Tumor purity for each sample was calculated using the ESTIMATE algorithm and subsequently included as a covariate in the statistical model for inter-group comparative analyses. To investigate the immune status of HCC patients, the CIBERSORT algorithm from “immunedeconv” (v 2.0.4) [18] was applied to assess the infiltration levels of 22 immune cell types in the high- and low-expression groups from TCGA-HCC. CIBERSORT analysis was performed on a log_2_-normalized expression matrix with 1000 permutations. Then, the difference in the infiltration score between the high- and low-expression groups was analyzed via Wilcoxon’s test with Benjamini–Hochberg (BH) correction for multiple testing (FDR < 0.05). Using the TIMER method, immune infiltration data were processed via the TIMER2.0 web server (https://compbio.cn/timer2/), and the results corroborated the significantly differential immune cells identified in our study. Additionally, Spearman’s correlation analysis was conducted to examine the relationship between CDK7 and the differential immune cell types using psych (v 2.2.9) [19], with BH correction applied using the criteria |cor| > 0.30 and FDR < 0.05.

### 2.5 Gene Mutation Analysis of CDK7

The *CDK7* mutation status in patients with HCC from TCGA-HCC was assessed using cBioPortal for Cancer Genomics (http://www.cbioportal.org/). Additionally, we investigated the relationship between *CDK7* mRNA expression and copy number alterations, and the results were visualized in a box plot.

### 2.6 CDK7 Drug Sensitivity Analysis

In an attempt to ascertain the correlation between CDK7 expression and drug sensitivity, the CellMiner database (http://discover.nci.nih.gov/cellminer/home.do) was employed to procure the NCI-60 chemical activity data and the associated RNA-seq expression dataset. Following this, Spearman correlation analysis was performed to calculate the association coefficient between CDK7 and drug, with a screening criterion of *p* < 0.05, and the results were displayed via “ggplot” (v 3.3.5) [20].

### 2.7 Clinical Characteristics Analysis

To investigate whether CDK7 expression affected the clinicopathological progression and prognosis of patients with HCC, clinical characteristics were included in this study, including age (>60 years vs. ≤60 years), sex (female vs. male), T stage (T1–T4), N stage (N0–N1), and M stage (M0–M1). Subsequently, the Wilcoxon test was employed to analyze the differences in CDK7 expression among different subgroups based on these clinical characteristics.

### 2.8 Identification of Intersection Genes

Differential expression analysis was performed to identify differentially expressed genes (DEGs) for high vs. low expression (DEGs 1) and HCC vs. control (DEGs 2) comparisons in the TCGA-HCC dataset via the DESeq2 package (v 1.34.0) (v 1.34.0) [21].

The screening criteria were |log_2_FC| ≥ 1 and adj. *p* < 0.05. Meanwhile, volcano maps and heatmaps were applied to display the DEGs 1 and DEGs 2 using ggplot2 and Pheatmap (v 0.7.7) [22], respectively. Next, the intersecting genes were identified by overlapping DEGs 1 and DEGs 2.

### 2.9 Development and Validation of the Risk Model

Based on the intersection genes, the univariate cox analysis was performed to screen candidate genes via “survival” (v 3.3-1) [23] in the TCGA-HCC dataset, with thresholds of a hazard ratio (HR) significantly deviating from 1 (HR ≠ 1) and *p* < 0.05 (i.e., HR > 1 indicates high risk, HR < 1 indicates low risk). The results were presented using forestplot (v 2.0.1) [24].) Based on the candidate genes, the least absolute shrinkage and selection operator (LASSO) algorithm was utilized to select prognostic genes via glmnet (v 4.1-2) [25]. The smallest lambda value was selected as the optimal cutoff, and 10-fold cross-validation was employed. Subsequently, a risk model was constructed using the prognostic genes. The risk score was calculated using the following formula:

$$\mathrm{risk}\mathrm{score}=\sum_{i=1}^{n}\left({\mathrm{coef}}_{i}*X_{i}\right)$$

where coef is the prognostic gene coefficient, and x represents the expression of prognostic genes. Moreover, to evaluate and validate this risk model, patients with HCC in the TCGA-HCC and GSE14520 datasets were divided into high- and low-risk cohorts based on the median risk score. Furthermore, KM curves were plotted using survminer (v 0.4.9) [26] (*p* < 0.05, log-rank test using the survdiff method) in the TCGA-HCC and GSE14520 datasets. Simultaneously, the risk score and survival time distributions of the two risk cohorts were plotted to evaluate the prognostic value of the risk model in both datasets. Clinical net benefit was assessed via decision curve analysis (DCA) calibration curves generated using the riskRegression package (2025.9.17) to compare the risk model with TNM staging and simple strategies. The C-index was determined using the concordance.indexfunction from survcomp (v 1.58.0), and statistical significance was evaluated using standard errors, with confidence intervals (CIs) estimated using the Noether method.

### 2.10 Independent Prognostic Analysis and Clinical Characteristics Analysis

To screen independent prognostic factors for HCC, the risk score and clinical characteristics (age, sex, and T, N, and M stages) were integrated for subsequently analysis. Then, univariate and multivariate Cox analyses (*p* < 0.05) were performed in TCGA-HCC. Both univariate and multivariate analyses included Schoenfeld residual testing.

To further validate the reliability of the risk model as a clinical prediction tool for patients with HCC, the survival (v 3.3-1) and survminer (v 0.4.9) packages were separately utilized to explore the survival difference in clinical characteristics between high- and low-risk cohorts from TCGA-HCC. The clinical characteristics included age (>65 vs. ≤65), sex (male vs. female), T stage (T0/1 vs. T2 vs. T3 vs. T4), N stage (N0), and M stage (M0 vs. M1). Later, Wilcoxon’s rank-sum test was employed to analyze the difference in the risk score based on various clinical characteristics (*p* < 0.05). The results were presented as a box line diagram.

### 2.11 Mutation Landscape Analysis and Correlation Analysis

Mutation frequencies were analyzed in the high- and low-risk cohorts using maftools [27]. Immediately, the top 20 genes, based on mutation frequency, were selected in each cohort, and waterfall plots were created to visualize the results. Similarly, we executed gene mutation analysis of the prognostic genes.

To explore the associations between CDK7 and prognostic genes, Spearman’s correlation analysis was executed using “psych” with |cor| > 0.30 and *p* < 0.05 as the screening criteria.

### 2.12 Cell Lines and Culture Conditions

The murine hepatoma cell lines HEPA1-6 (CRL-1830) and Hepa1c1c7 (CRL-2026) were sourced from the National Collection of Authenticated Cell Cultures (Shanghai, China). Murine cell line identity was confirmed by STR analysis. Cells were cultured in high-glucose Dulbecco’s Modified Eagle’s Medium (DMEM; Servicebio, Wuhan, China) supplemented with 10% fetal bovine serum (FBS; Servicebio) and 1% penicillin–streptomycin (Servicebio). Cells were plated at 10,000 cells per well in growth medium on a 96-well plate and incubated at 37°C with 5% CO_2_ for 24 h. Cells were routinely tested for mycoplasma contamination using a mycoplasma detection kit (Beyotime, Shanghai, China) and confirmed to be negative.

### 2.13 Lentivirus Infection and Plasmids Transfection

For stable *CDK7* knockdown or overexpression, lentiviral particles carrying short hairpin RNA (shRNA) targeting CDK7 (sh-CDK7), negative-control shRNA (sh-NC), or *CDK7* overexpression sequences were designed and constructed by RiboBio Corporation (Guangzhou, China). For transient knockdown, chemically synthesized small interfering RNA (siRNA) targeting *CDK7* (si-CDK7) and negative-control siRNA (si-NC) were obtained from the same company. The target sequences and the sense/antisense strand sequences are detailed in Supplementary Table S1. To avoid confusion, siRNA was used for *in vitro* transient knockdown and *in vivo* intratumoral injection, whereas lentivirus was used to establish stable *in vitro* knockdown or overexpression cell lines.

For transient transfection, cells were seeded at a density of 2 × 10^5^ cells per well in six-well plates and cultured to 60%–80% confluence. Transfection of siRNAs (50 nM) or overexpression plasmids (1 μg) was performed using Lipofectamine 3000 reagent (Thermo Fisher Scientific, Waltham, MA, USA) according to the manufacturer’s protocol, as previously described [28]. After 72 h of transfection, cells were harvested for subsequent experiments.

For lentiviral transduction, cells were incubated with lentiviral particles at a multiplicity of infection of 10 in the presence of 8 μg/mL polybrene (Sigma-Aldrich, St. Louis, MO, USA) for 24 h. The medium was then replaced with fresh complete medium, and stable transductants were selected by treatment with puromycin (2 μg/mL; InvivoGen, San Diego, CA, USA) for 7 days. Knockdown or overexpression efficiency was confirmed by western blotting.

### 2.14 Western Blot

Western blotting was performed as previously specified [29]. In brief, cells were lysed in radioimmunoprecipitation assay buffer (Thermo Fisher Scientific) supplemented with protease and phosphatase inhibitors (Thermo Fisher Scientific). Protein concentrations were determined using a bicinchoninic acid protein assay kit (Thermo Fisher Scientific). Equal amounts of total protein (30 μg per lane) were separated by 10%–12% sodium dodecyl sulfate-polyacrylamide gel electrophoresis at 100 V for 2 h and then transferred onto polyvinylidene difluoride 10–12% membranes (MilliporeSigma, Burlington, MA, USA) using a transfer system (Bio-Rad, Hercules, CA, USA). Membranes were blocked with 5% nonfat milk in Tris-buffered saline containing 0.1% Tween-20 (TBST) for 1 h at room temperature and then incubated with the following primary antibodies overnight at 4°C: rabbit anti-CDK7 (catalog No. 27027-1-AP, 1:1000 dilution), rabbit anti-p65 (catalog No. 80979-1-RR, 1:1000), rabbit anti–phospho-p65 (p-p65, catalog No. 82335-1-RR, 1:1000), and mouse anti–beta-actin (catalog No. 66009-1-Ig, 1:1000). All primary antibodies were purchased from Proteintech (Rosemont, IL, USA). After washing three times with TBST, membranes were incubated with horseradish peroxidase (HRP)-linked secondary antibodies (all from Proteintech), including HRP-linked anti-rabbit IgG (catalog No. SA00001-2, 1:1000) and HRP-linked anti-mouse IgG (catalog No. SA00001-1, 1:1000), for 1 h at room temperature. Detailed information is provided in Supplementary Table S2. Protein signals were detected using an enhanced chemiluminescence (ECL) reagent (SignalFire™ ECL Reagent, Cell Signaling Technology, Danvers, MA, USA) and visualized using an Amersham Imager 600 (GE Healthcare). Band intensities were quantified using ImageJ software (National Institutes of Health, Bethesda, MD, USA), with beta-actin serving as the loading control. Full, uncropped western blot images are provided in Supplementary File S1.

### 2.15 Transwell Migration and Invasion Assay

The Transwell migration assay was performed using 8-μm pore-size Transwell plates (NEST, Wuxi, China). Cells were suspended in 200 μL of serum-free DMEM (Servicebio) at a density of 1 × 10^5^ cells per well and seeded into the upper chambers of 24-well plates. The lower chambers were filled with 600 μL of DMEM containing 20% FBS. After incubation at 37°C for 24 h, nonmigrating cells on the upper membrane surface were gently removed with a cotton swab. Migrating cells on the lower membrane surface were fixed with 100% methanol for 30 min and stained with 0.5% crystal violet solution for 30 min at room temperature. Stained cells were photographed and counted in five randomly selected fields under a light microscope (Olympus Corporation, Tokyo, Japan).

For the invasion assay, the upper chambers were precoated with Matrigel (Corning, New York, NY, USA) diluted 1:15 with serum-free DMEM. Then, 60 μL of the diluted Matrigel was added to each upper chamber and incubated at 4°C for 4–6 h to allow solidification. The remaining steps were identical to those of the migration assay. All experiments were performed independently three times, and the counting was performed by an investigator blinded to the experimental groups.

### 2.16 Scratch Assay and Colony Formation Assay

For the scratch assay, cells were seeded in six-well plates at a density of 5 × 10^5^ cells per well and cultured until reaching approximately 90% confluence. A linear scratch was created across the cell monolayer using a sterile 200-μL pipette tip. Debris was removed by washing with phosphate-buffered saline (PBS), and cells were cultured in serum-free medium. Wound closure was photographed at 0 and 24 h using a cell imaging system (Olympus Corporation). The wound-healing rate was calculated as the percentage of the initial wound area that was closed. Three independent experiments were performed, and five random fields were quantified per experiment.

The colony formation assay was performed as previously described [30]. Briefly, cells were seeded in six-well plates at a density of 1 × 10^3^ cells per well and cultured at 37°C in 5% CO_2_ for 7–10 days, with medium replacement every 2–3 days. When visible colonies appeared, cells were fixed with 100% methanol for 30 min and stained with 0.5% crystal violet solution for 30 min at room temperature. Colonies containing more than 50 cells were counted under a microscope. The assay was performed in triplicate.

### 2.17 Mouse Experiments

All animal experiments were approved by the Animal Ethics and Welfare Committee of Hunan Provincial People’s Hospital (The First Affiliated Hospital of Hunan Normal University; Ethics approval No. [2024]-39) and conducted in accordance with the National Institutes of Health Guide for the Care and Use of Laboratory Animals.

Male BALB/c nude mice (4 weeks old; body weight, 18–20 g) were obtained from Jiangsu Kingsray Biotechnology Co., Ltd. (Nanjing, China). Mice were housed under specific pathogen-free conditions with a 12-h/12-h light/dark cycle, temperature of 22.0 ± 2.0°C, humidity of 50.0% ± 10.0%, and free access to food and water. No animals were excluded from the analysis unless they met predefined exclusion criteria (e.g., severe weight loss > 25.0% or spontaneous death). Sample size was estimated using a power analysis (α = 0.05, β = 0.2) based on previous pilot data (120.0 ± 25.0 mm^3^ tumor volume at baseline), yielding five mice per group. Mice were randomly assigned to two groups (n = 5 per group) using a computer-generated random number table, namely the si-NC and si-CDK7 groups. Tumor cell inoculation and subsequent measurements were performed by an investigator blinded to the group allocation.

Approximately 1 × 10^6^ HEPA1-6 cells suspended in 100 μL of PBS were inoculated subcutaneously into the right flank of each mouse. When the tumor volume reached approximately 100 mm^3^, intratumoral injections of siRNA solution (20 μL per mouse, containing siRNA and Lipofectamine 3000 at a 1:1 volume ratio) were administered every 3 days for 4 weeks. Tumor dimensions (length and width) were measured every 3 days using a digital caliper, and tumor volume was calculated using the formula: V = (length × width^2^)/2. At the end of the 4-week period, mice were euthanized via CO_2_ inhalation, and tumors were excised and weighed. Tumor growth curves were plotted, and final tumor weights were compared between the two groups using an unpaired two-tailed Student’s *t*-test. For tumor growth curves over time, a two-way repeated measures ANOVA followed by Bonferroni’s post-hoc test was applied. All statistical tests were two-sided, and a *p* < 0.05 was considered statistically significant. All animal procedures were performed in accordance with institutional ethical guidelines.

### 2.18 Statistical Analysis

Statistical analyses were performed using R software (version 4.2.3, R Foundation for Statistical Computing, Vienna, Austria) and GraphPad Prism 8.0 (GraphPad Software, Boston, MA, USA). For bioinformatics analyses, differential expression between two groups was assessed using the Wilcoxon rank-sum test. KM survival curves were generated using the survival package, and the log-rank test was used to compare survival distributions. For LASSO regression and Cox proportional hazards models, the proportional hazards assumption was verified using Schoenfeld residuals. Receiver operating characteristic (ROC) curves were generated using the timeROC package, and the area under the ROC curve (AUC) and its 95% CI were calculated using inverse probability of censoring weighting. For multiple comparisons in immune infiltration analysis, BH FDR correction was applied, and FDR < 0.05 was considered statistically significant. All analysis codes are provided in Supplementary File S2.

For *in vitro* and *in vivo* experiments, data are presented as the mean ± standard deviation (SD) of at least three independent biological replicates. Comparisons between two groups were performed using the two-tailed Student’s *t*-test (for normally distributed data) or the Mann–Whitney U test (for nonnormally distributed data). Comparisons among multiple groups were performed using one-way analysis of variance followed by Tukey’s post hoc test. *p* < 0.05 was considered statistically significant. All statistical tests were two-sided, and no adjustments for multiple testing were applied to the *in vitro* and *in vivo* experiments unless otherwise specified.

## 3 Results

### 3.1 CDK7 Was Mainly Enriched in Complement and Coagulation Cascades

**Figure 1:**
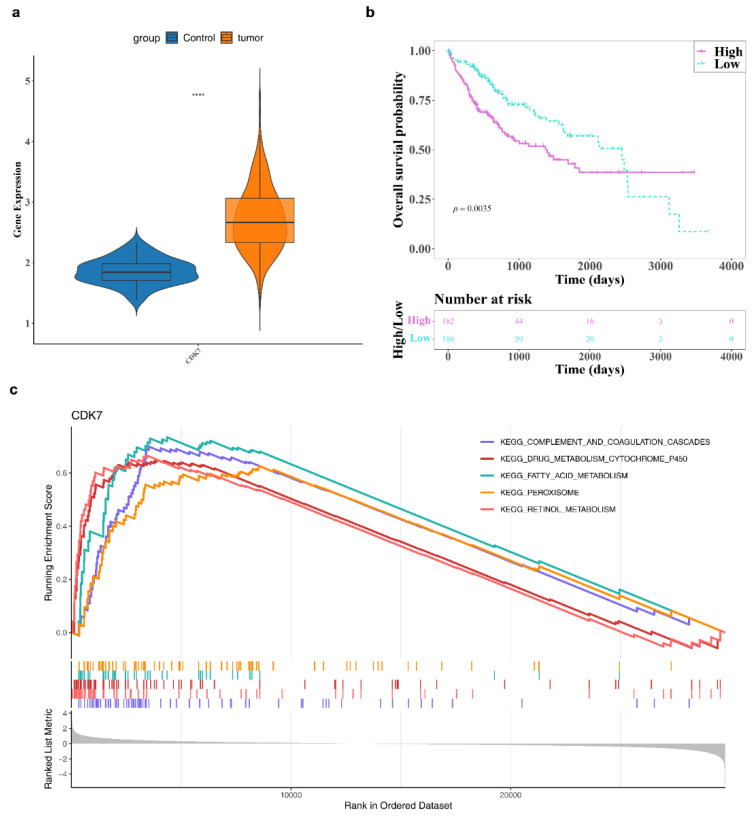
Analysis of differential *CDK7* expression in HCC. (**a**) *CDK7* is upregulated in HCC tumors (n = 374) versus normal tissues (n = 50) from TCGA-LIHC (Wilcoxon’s test, ****p < 0.0001). (**b**) Patients with low *CDK7* expression (n = 187, median split) exhibit better overall survival than those with high expression (n = 187) in TCGA-LIHC (log-rank test, *p* = 0.0035). (**c**) GSEA reveals the top five KEGG pathways enriched in the high *CDK7* expression group (NES > 1, q < 0.25). The leading pathway is presented. (Abbreviations: CDK7, Cyclin-Dependent Kinase 7; HCC, Hepatocellular Carcinoma; TCGA-LIHC, The Cancer Genome Atlas Liver Hepatocellular Carcinoma; GSEA, Gene Set Enrichment Analysis; KEGG, Kyoto Encyclopedia of Genes and Genomes).

Based on gene expression analysis, *CDK7* expression was higher in HCC samples from TCGA-HCC (Fig. 1a). Additionally, KM curve analysis indicated that the overall survival probability was higher in the low-expression group (*p* = 0.0035; Fig. 1b). To gain preliminary insights into the biological processes associated with *CDK7* expression in HCC, GSEA was performed. GSEA revealed that *CDK7* was significantly enriched in several Kyoto Encyclopedia of Genes and Genomes (KEGG) pathways. Specifically, the top five KEGG pathways included “complement and coagulation cascades,” “drug metabolism cytochrome P450,” “fatty acid metabolism,” “peroxisome,” and “retinol metabolism” (Fig. 1c).

### 3.2 The 6 Differential Immune Cells Were Detected

**Figure 2:**
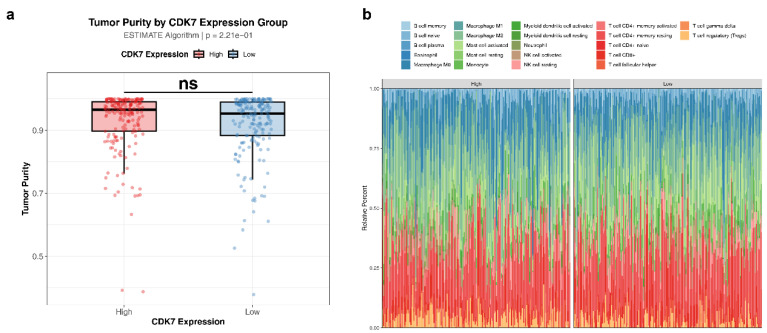

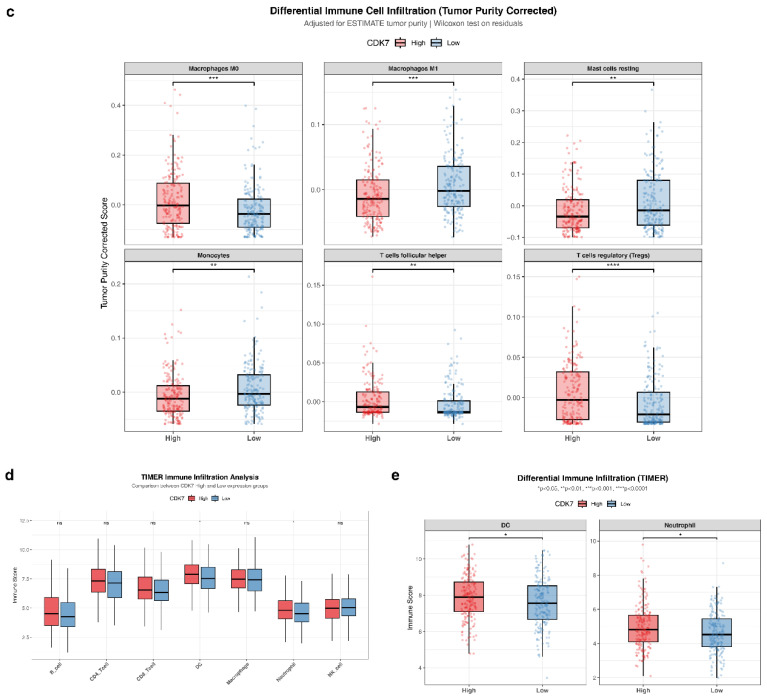
Immune microenvironment analysis of *CDK7* in HCC. (**a**) Comparison of tumor purity between the high- and low-expression groups (*p* = 0.221). (**b**) Overview of the relative abundance of 22 immune cell types in the two groups. (**c**) Six immune cell types exhibited significant differences between the groups, including M0/M1 macrophages, resting mast cells, monocytes, and follicular helper/regulatory T cells (FDR < 0.05). (**d**,**e**) Validation using the TIMER algorithm, confirming the higher infiltration of CD4^+^ T cells, B cells, and macrophages in the high-expression group. *p < 0.05; **p < 0.01; ***p < 0.001; ****p < 0.0001; ns: no significant. (Abbreviations: CDK7, Cyclin-Dependent Kinase 7; HCC, Hepatocellular Carcinoma; TIMER, Tumor Immune Estimation Resource).

Tumor purity did not differ between the two groups (*p* = 0.221; Fig. 2a). Fig. 2b displays the immune infiltration status of 22 immune cell types in the high- and low-expression groups. Six differential immune cell types were detected, including M0 macrophages, M1 macrophages, resting mast cells, monocytes, follicular helper T cells, and regulatory T cells (Fig. 2c). Immune infiltration analysis via TIMER indicated significantly higher infiltration of CD4^+^ T cells, B cells, and macrophages in the high-*CDK7* expression group, reinforcing the stability of our observations (Fig. 2d,e). However, Spearman correlation analysis demonstrated that the continuous expression level of CDK7 partially exhibited a linear correlation with the infiltration scores of these specific cell populations (|r| < 0.30, *p* < 0.05; Supplementary Table S3). This suggests in an exploratory manner that although CDK7 expression is associated with a distinct immune contexture, the relationship might not be direct and could be mediated by other downstream factors.

### 3.3 The Gene Mutation and Drug Sensitivity Analyses of CDK7 Might Be Helpful for Exploring the Mechanism of HCC

**Figure 3:**
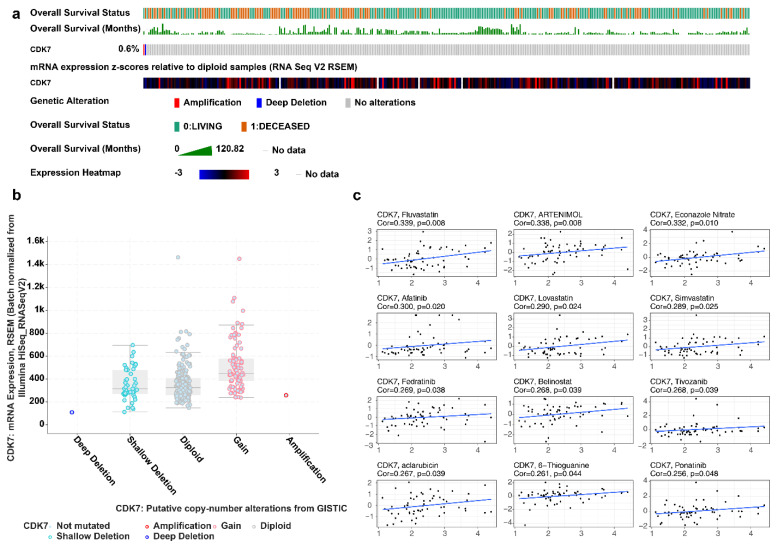
Gene mutation and drug sensitivity analyses of *CDK7* in HCC. (**a**) Oncoprint reveals the extremely low frequency of genomic alterations (0.6%, 2/374 samples) in the CDK7 gene across the TCGA-HCC cohort. (**b**) *CDK7* mRNA expression grouped by the copy number alteration status in TCGA-HCC. Gain is associated with higher expression (Wilcoxon’s test). (**c**) The association between CDK7 expression and drug sensitivity. The Spearman correlation coefficient (r) and nominal *p*-value are labeled. (Abbreviations: CDK7, Cyclin-Dependent Kinase 7; HCC, Hepatocellular Carcinoma; TCGA-HCC: The Cancer Genome Atlas Hepatocellular Carcinoma.).

Gene mutation analysis revealed that *CDK7* exhibited an extremely low frequency of alterations in HCC samples (0.6%), with only two samples displaying amplification and deep deletion mutations (Fig. 3a). This suggests that *CDK7* is unlikely to be a mutation-driven oncogene in HCC, and its oncogenic role is more likely exerted at the level of transcriptional or expression regulation. Furthermore, Fig. 3b indicates that the gain mutation type was associated with the highest mRNA expression of *CDK7*. This observation suggested that as the copy number of *CDK7* increases, its mRNA expression also gradually rises. Additionally, drug sensitivity analysis identified 12 drugs with significant correlations with CDK7, including fluvastatin (r = 0.339, *p* = 0.008), artenimol (r = 0.338, *p* = 0.008), and econazole nitrate (r = 0.332, *p* = 0.010; Fig. 3c).

### 3.4 Differential Expression of CDK7 across Clinical Subgroups

**Figure 4:**
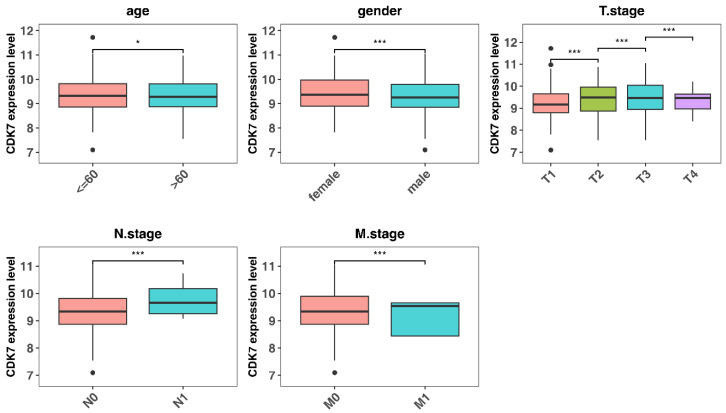
Comparison of *CDK7* expression in the TCGA-HCC cohort stratified by gender, age, and AJCC T, N, and M stages. Each box represents the interquartile range, and the horizontal line inside the box denotes the median. The number of samples (n) for each subgroup is indicated in parentheses on the *x*-axis (Supplementary Table S4). *p*-values were calculated using the Wilcoxon rank-sum test for two-group comparisons (nominal, uncorrected). *p < 0.05; ***p < 0.001. (Abbreviations: CDK7, Cyclin-Dependent Kinase 7; TCGA-HCC: The Cancer Genome Atlas Hepatocellular Carcinoma; AJCC, American Joint Committee on Cancer).

To descriptively explore the relationship between *CDK7* expression and clinicopathological features, we compared its levels across different clinical subgroups in the TCGA-HCC cohort. *CDK7* expression exhibited significant differences among different clinical characteristic subgroups (*p* < 0.05). Specifically, *CDK7* expression was significantly higher in female patients than in male patients, higher in individuals aged ≤ 60 years than in those aged > 60 years, and higher in stages N1 and M0 than in stages N0 and M1 stages, respectively (*p* < 0.05; Fig. 4). Additionally, *CDK7* expression was significantly higher in stage T2 than in stages T1 and T3 (Fig. 4).

### 3.5 Ninety-Eight Intersecting Genes Were Identified

**Figure 5:**
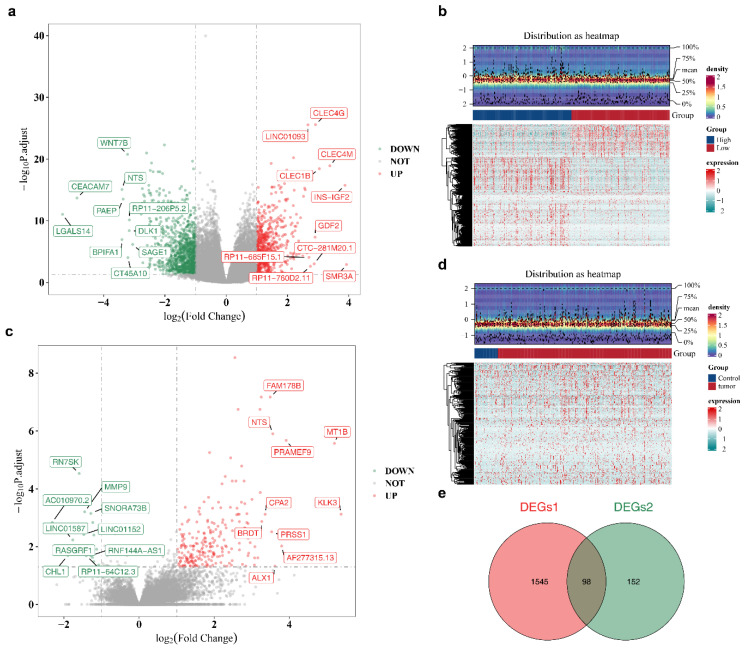
Differential expression analysis was conducted to identify the intersecting genes in the TCGA-HCC dataset. (**a**,**b**) Volcano plot (**a**) and heatmap (**b**) of DEGs 1 between the high- and low-expression groups in TCGA-HCC (|log_2_FC| ≥ 1, adj. *p* < 0.05). (**c**,**d**) Volcano plot (**c**) and heatmap (**d**) of DEGs 2 between HCC tumors and normal liver tissues in TCGA-HCC (|log_2_FC| ≥ 1, adj. *p* < 0.05). (**e**) Venn diagram revealing the overlap of 98 genes between DEGs 1 and DEGs 2. (Abbreviations: TCGA-HCC: The Cancer Genome Atlas Hepatocellular Carcinoma; DEGs: Differentially Expressed Genes.).

Differential expression analysis identified 1643 DEGs 1 (661 upregulated and 1032 downregulated genes; Fig. 5a,b) between the high- and low-expression groups and 250 DEGs 2 (233 upregulated and 17 downregulated genes; Fig. 5c,d) between HCC and control samples in TCGA-HCC. Subsequently, 98 intersecting genes were selected by overlapping the 1643 DEGs 1 and 250 DEGs 2 (Fig. 5e). These overlapping genes represent a set of genes co-associated with both *CDK7* expression and hepatocellular carcinogenesis.

### 3.6 The Risk Model Had a Reliable Predictive Ability

**Figure 6:**
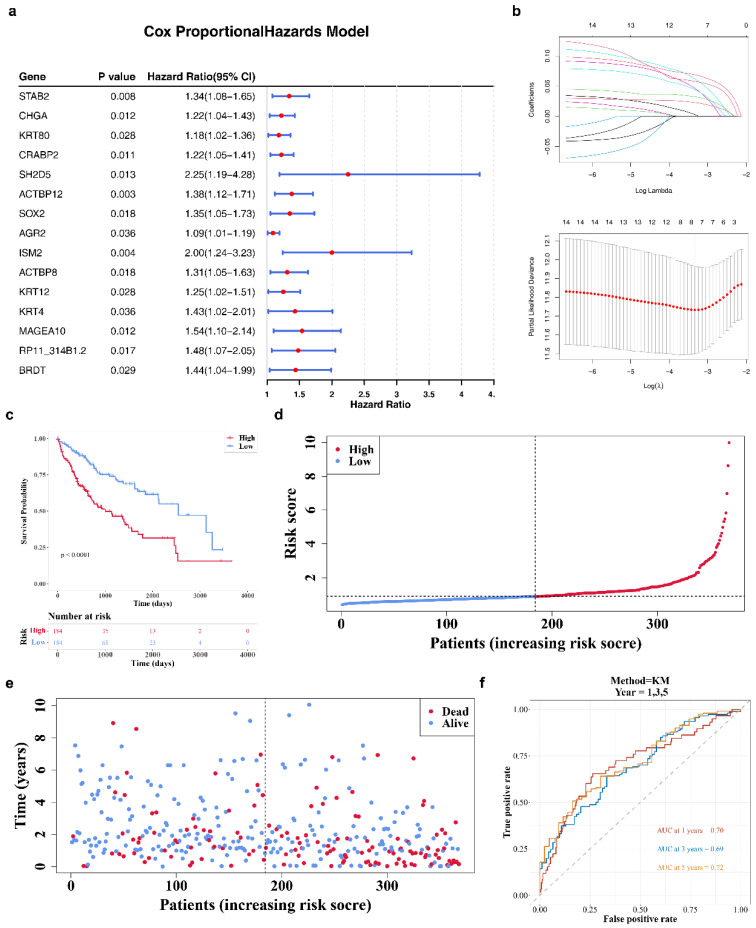
Establishment of the risk model and evaluation of its predictive ability. (**a**) Among the 98 intersecting genes, 15 genes were selected by univariate Cox analysis. (**b**) Eight genes were further identified as prognostic genes by LASSO regression. (**c**–**e**) Patients with HCC were classified into 184 high-risk and 184 low-risk samples. KM curve analysis indicated that high-risk patients had a lower survival probability, a higher risk score, and shorter survival time than low-risk patients. (**f**) The ROC curves for the training set at 1 (AUC = 0.70, 95% CI = 0.63–0.78), 3 (AUC = 0.69, 95% CI = 0.62–0.76), and 5 years (AUC = 0.72, 95% CI = 0.63–0.80). (Abbreviations: LASSO: Least Absolute Shrinkage and Selection Operator; HCC, Hepatocellular Carcinoma; KM, Kaplan-Meier; ROC, Receiver Operating Characteristic; AUC, Area Under the Curve; CI, Confidence Interval).

**Figure 7:**
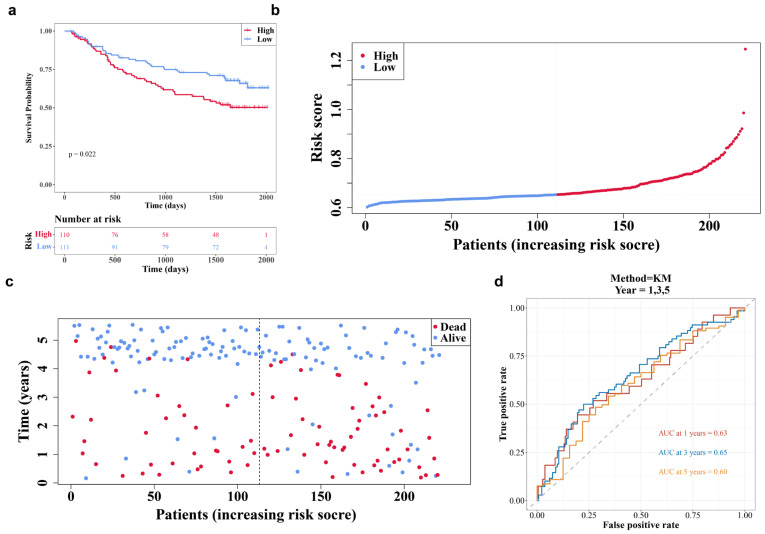
Evaluation of the risk model’s predictive ability in GSE14520. (**a**–**c**) The results of KM curve, risk curve, scatter diagram, and ROC curve analyses in the GSE14520 dataset. (**d**) The ROC curves for the training set at 1 (AUC = 0.63, 95% CI = 0.51–0.74), 3 (AUC = 0.65, 95% CI = 0.57–0.73), and 5 years (AUC = 0.60, 95% CI = 0.48–0.72). (Abbreviations: KM, Kaplan-Meier; ROC, Receiver Operating Characteristic; AUC, Area Under the Curve; CI, Confidence Interval).

Among the 98 intersecting genes, 15 genes remained significant in univariate Cox analysis (*p* < 0.05), including *SH2D5*, *CHGA*, and *ACTBP12* (Fig. 6a). Subsequently, eight genes were further identified as prognostic genes by LASSO (lambda_min_ = 0.036), namely *CHGA*, *SH2D5*, *ACTBP12*, *SOX2*, *AGR2*, *ISM2*, *KRT12*, and *BRDT* (Fig. 6b). Using these eight prognostic genes, a risk model was developed. In TCGA-HCC, patients with HCC were classified into high- (n = 184) and low-risk (n = 184) groups. KM curves revealed that high-risk patients had a lower survival probability (Fig. 6c). Additionally, the high-risk cohort exhibited higher risk scores and shorter survival times (Fig. 6d,e). The ROC curve demonstrated that the AUCs at 1, 3, and 5 years were 0.70, 0.69, and 0.72, respectively. Thus, these values exceeded 0.6 throughout the survival period, suggesting moderate predictive ability (Fig. 6f). Similarly, in GSE14520, patients with HCC were classified high- (n = 110) and low-risk groups (n = 111). The results of the KM curve, risk curve, scatter diagram, and ROC curve analyses in the GSE14520 dataset aligned with those in the TCGA-HCC dataset, with the risk model retaining acceptable, though slightly attenuated, predictive performance in this independent dataset (Fig. 7a–d).

**Figure 8:**
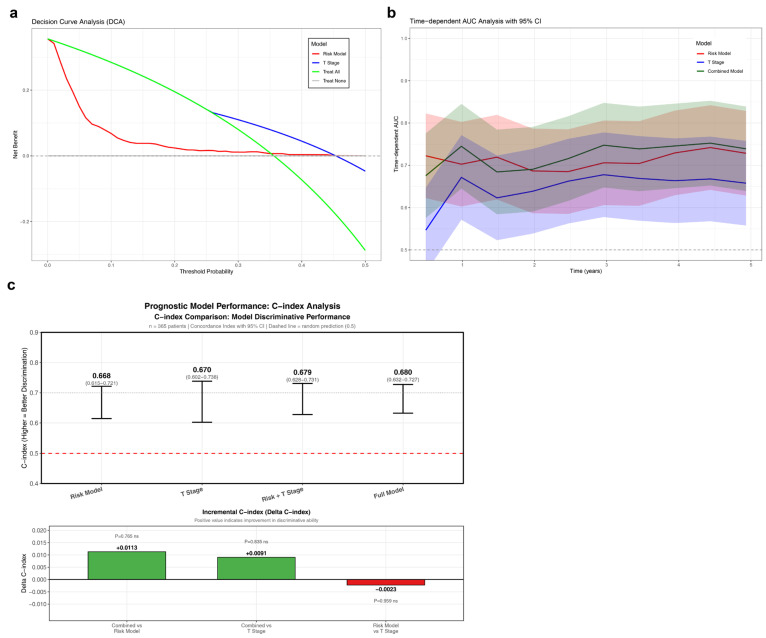
Clinical utility and predictive performance of the risk model. (**a**) DCA demonstrated that the risk model provided greater clinical net benefit than TNM stage or simple strategies for threshold probabilities of 5%–45%. (**b**) Time-dependent AUC curves indicate that the risk model outperformed T stage alone (*p* < 0.01) at all time points, with the combined model (risk score + T stage) exhibiting the best performance. A mild decline in the AUC over time was observed. (**c**) Comparison of C-indices among different models. ns: not significant. (Abbreviations: DCA, Decision Curve Analysis; TNM, Tumor, Node, Metastasis).

When the threshold probability was 5%–45%, the risk model displayed greater clinical net benefit than TNM stage or simple strategies (Fig. 8a). The biggest difference versus the T-stage model appeared at 15%–35%. The risk model had a significantly better AUC than T stage alone at all time points (*p* < 0.01). The combined model (risk score + T stage) performed best, although all AUCs declined slightly over time (Fig. 8b, Supplementary Table S5). The 1-year AUC was 0.742, highlighting good short-term predictive performance. The combined model also had the highest C-index (0.679), but this was not a statistically significant improvement (Fig. 8c, Supplementary Table S6). Adding age and sex to the full model did not clearly improve the C-index.

### 3.7 Six Prognostic Genes Harbored Missense Mutations

**Figure 9:**
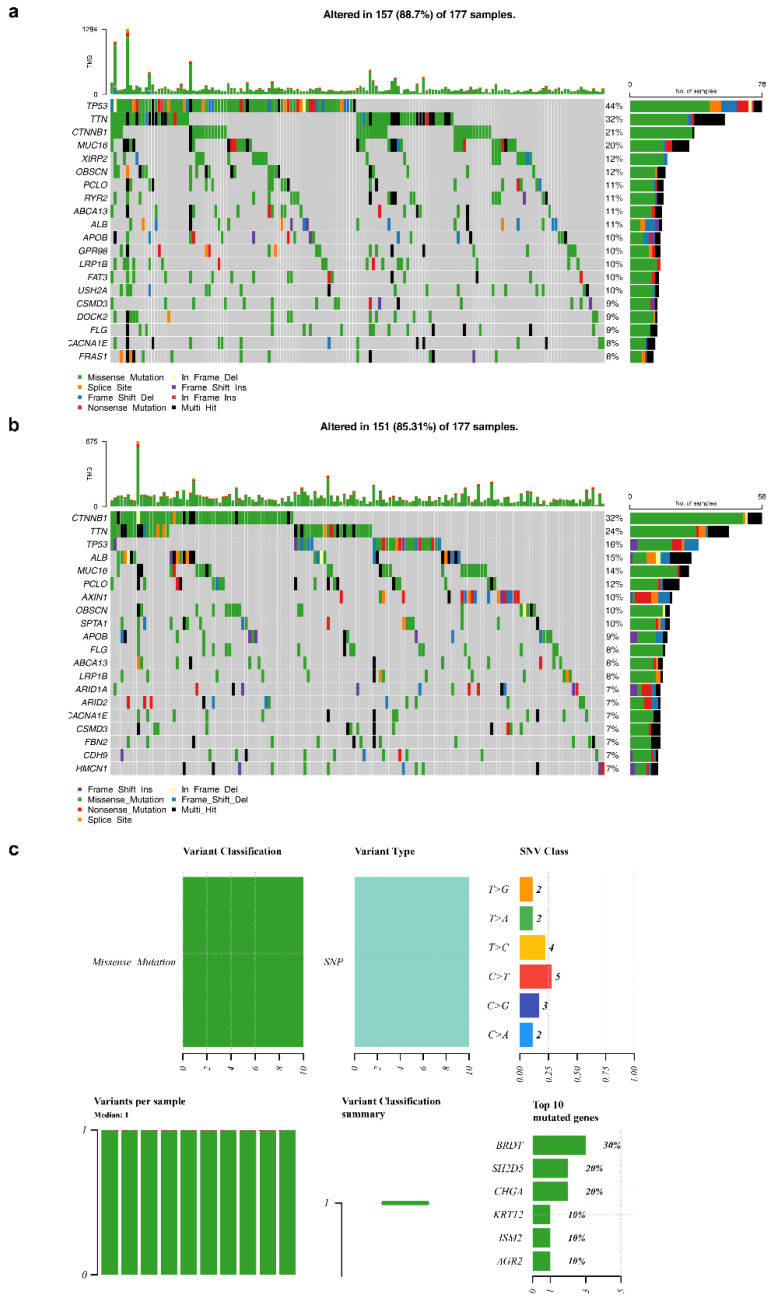
Mutation analysis in the two risk cohorts. (**a**,**b**) Mutation analysis of *TP53* and *CTNNB1*. (**c**) Six prognostic genes harbored missense mutations.

Mutation analysis in the two risk cohorts indicated that *TP53* was the most frequently mutated gene in the high-risk group (44%), whereas *CTNNB1* was the most frequently mutated gene in the low-risk group (32%; Fig. 9a,b). The mutation types included missense mutations, splice site, and multiple-hit mutations. Additionally, six prognostic genes harbored missense mutations (e.g., C>T, T>C, C>G): *BRDT*, *SH2D5*, *CHGA*, *KRT12*, *ISM2*, and *AGR2*. Additionally, these six prognostic genes harbored single-nucleotide polymorphisms (Fig. 9c). This suggests that missense mutations in these signature genes might alter protein function, potentially contributing to the aggressive phenotype identified by our risk model.

### 3.8 Identification of Independent Prognostic Factors and Survival Analysis Based on the Risk Score and Clinical Characteristics

**Figure 10:**
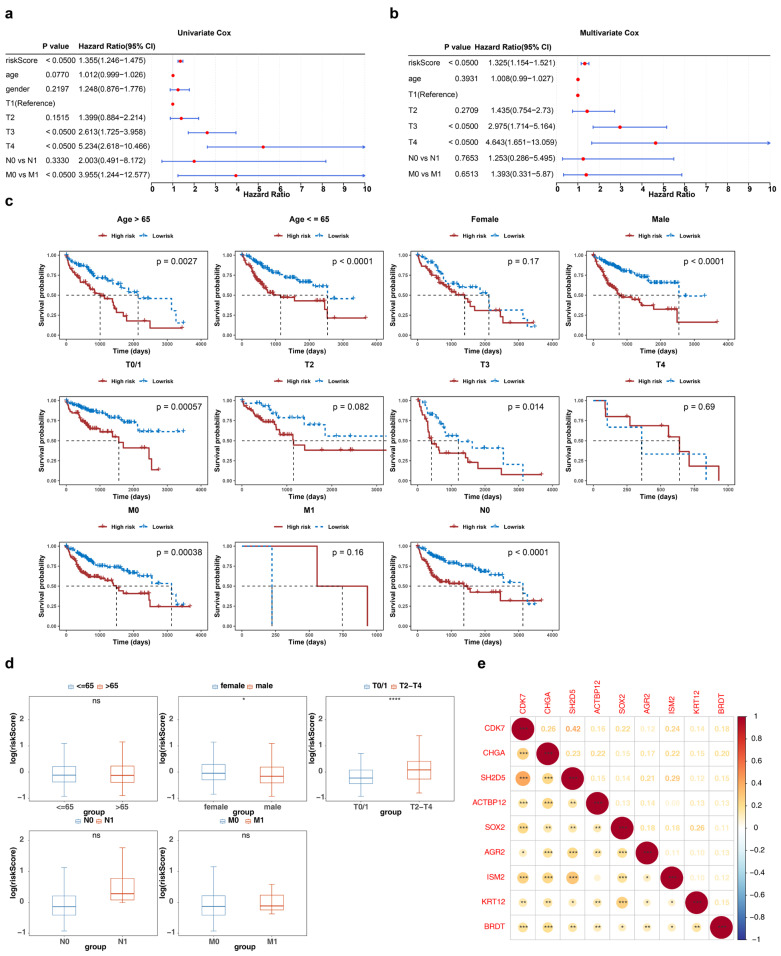
The prognostic value of the risk score in different subgroups based on clinical features. (**a**) Univariate Cox analysis identified the risk score, T stage and M stage as significant independent prognostic factors. (**b**) Multivariate Cox analysis identified the risk score and T stage as independent prognostic factors. (**c**) Significant survival differences were observed according to clinical characteristics between the high- and low-risk groups. (**d**) The prognostic value of the risk score in different subgroups based on clinical features. ns, not significant; *p < 0.05; ****p < 0.0001 (**e**) Spearman’s correlation analysis revealed a significant positive correlation between *CDK7* and *SH2D5*. *p < 0.05; **p < 0.01; ***p < 0.001.

Based on univariate Cox analysis, the risk score, T stage and M stage exhibited significant independent prognostic value (*p* < 0.05, unadjusted; Fig. 10a). Significant survival differences were observed between the high- and low-risk groups in subgroups stratified by age (>60 years, n = 195; ≤60 years, n = 173), sex (male, n = 249; female, n = 119), T stage (T1, n = 182; T2, n = 92; T3, n = 78; T4, n = 13), M stage (M0, n = 265; M1, n = 3), and N stage [N0, n = 250; N1 was excluded because of its limited sample size (n = 4); Fig. 10b]. The low-risk group exhibited a higher survival probability in these subgroups (*p* < 0.05, uncorrected for multiple testing). Two independent prognostic factors (risk score and T stage) were identified by multivariate Cox analysis (Fig. 10b).

Furthermore, significant survival differences were observed among clinical characteristic subgroups between the high and low-risk cohorts (Fig. 10c). Specifically, KM curves indicated notable disparities among the groups for age (>65 years vs. ≤65 years); sex (male patients); and stages T0/T1, T3, M0, and N0 (*p* < 0.05). Notably, the low-risk cohort exhibited a higher survival probability in these subgroups. In addition, the risk score predicted a significant difference in survival between female and male patients and between stages T0/T1 and T2–T4 (Fig. 10d).

Spearman’s correlation analysis revealed a significant positive correlation between *CDK7* and *SH2D5* (r = 0.42, *p* < 0.01; Fig. 10e), indicating an expression association, but this represents correlative evidence rather than mechanistic proof.

### 3.9 Validate the Role of CDK7 through In Vitro and In Vivo Experiments

**Figure 11:**
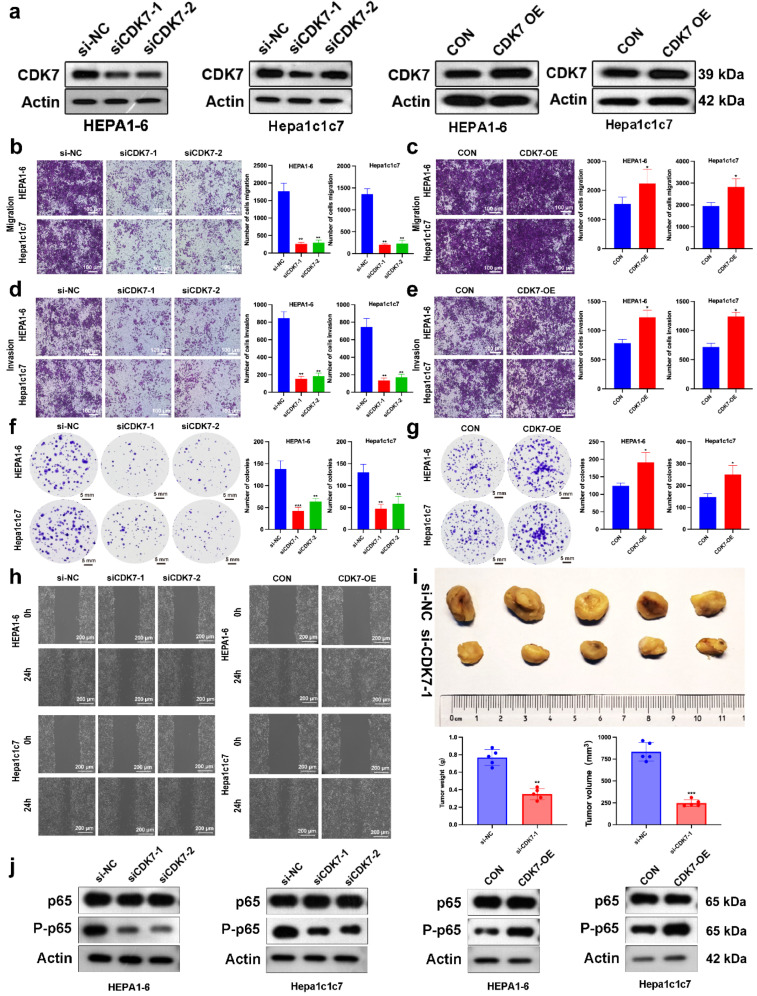
Validation of the functional role of CDK7 in HCC progression through *in vitro* and *in vivo* experiments. (**a**) Western blot confirming *CDK7* knockdown (si-CDK7) and overexpression (CDK7-OE) in HEPA1-6 and Hepa1c1c7 cells. Beta-actin was used as a loading control. Full, uncropped blots are provided in Supplementary File S1. (**b**,**c**) Transwell migration assays indicated that *CDK7* knockdown significantly reduced the migratory ability of HCC cells (**b**), whereas *CDK7* overexpression enhanced the migratory ability of HCC cells (**c**). Scale bar = 100 μm. (**d**,**e**) Transwell invasion assays (Matrigel-coated) demonstrating that *CDK7* knockdown significantly decreased the invasive capacity of HCC cells (**d**), whereas *CDK7* overexpression increased the invasive capacity of HCC cells (**e**). Scale bar = 100 μm. (**f**,**g**) Colony formation assays indicated that *CDK7* knockdown markedly suppressed the colony-forming ability of HCC cells (**f**), whereas CDK7 overexpression promoted the colony-forming ability of HCC cells (**g**). Scale bar = 5 mm. (**h**) Scratch assays illustrating that *CDK7* knockdown impaired wound closure, whereas CDK7 overexpression accelerated wound healing. Scale bar = 200 μm. (**i**) *In vivo* subcutaneous tumor growth assay in BALB/c nude mice. Mice were randomly assigned to the si-NC (negative control) and si-CDK7 groups (n = 5 per group). Intratumoral injections were administered every 3 days for 4 weeks. Tumor volumes were measured every 3 days (left panel), and final tumor weights were compared (right panel). *CDK7* knockdown significantly inhibited tumor growth. (**j**) Western blotting of total p65 and p-p65 after *CDK7* knockdown or overexpression, demonstrating that *CDK7* expression is associated with changes in p-p65 levels. Beta-actin served as a loading control. All quantitative data are presented as the mean ± SD of three independent biological replicates (n = 3 per group for *in vitro* assays; n = 5 mice per group for *in vivo* assays). For Transwell migration/invasion assays, cell counting was performed by an investigator blinded to the experimental groups. Statistical comparisons between two groups were performed using the two-tailed Student’s *t*-test (for normally distributed data) or the Mann–Whitney *U* test (for nonnormally distributed data). *p* < 0.05 was considered statistically significant. *p < 0.05; **p < 0.01; ***p < 0.001.

Finally, we validated the role of CDK7 in HCC through *in vitro* and *in vivo* experiments. First, we validated the expression of CDK7 in HCC cell lines by knocking down or overexpressing the gene via lentiviral transfection, followed by western blotting (Fig. 11a; the full uncropped blots are presented in Supplementary File S1). A series of functional assays were conducted. The cell migration experiments suggested that silencing *CDK7* significantly weakened the migration of HCC cell lines, whereas *CDK7* overexpression enhanced their migration (Fig. 11b,c). Invasion assays demonstrated that specific inhibition of *CDK7* significantly reduced cell invasiveness, whereas overexpression of *CDK7* led to increased invasion (Fig. 11d,e). Colony formation assays illustrated that following *CDK7* knockdown, HCC cells had significantly diminished ability to form colonies, with the opposite result observed upon *CDK7* overexpression (Fig. 11f,g). Additionally, we performed scratch assays, which similarly indicated that silencing *CDK7* significantly impaired the wound-healing capability of HCC cells, whereas *CDK7* overexpression improved wound-healing ability (Fig. 11h). Subsequently, we conducted subcutaneous tumorigenesis experiments in nude mice, revealing that *CDK7* inhibition significantly reduced liver cancer growth (Fig. 11i). These experiments confirmed that downregulation of CDK7 suppressed the proliferation, migration, and invasion of HCC cells; inhibited the malignant phenotype of HCC cells; and restrained *in vivo* growth. Finally, we performed western blotting to analyze pathways potentially related to CDK7, finding that *CDK7* significantly inhibited the expression of p-p65. Conversely, *CDK7* overexpression resulted in a marked increase in p-p65 expression (Fig. 11j). Our experiments showed that changes in CDK7 expression are associated with the phosphorylation level of RelA/p65, and this association correlates with the malignant phenotypes of HCC, though further studies are required to establish direct causality.

## 4 Discussion

HCC is a globally distributed malignancy [2]. As HCC research continues to deepen, new treatment strategies and management programs are constantly being developed [31,32]. By identifying risk factors early and developing appropriate preventive measures, the incidence of liver cancer can be effectively reduced, and survival rates can be improved [3,4]. Recent studies have indicated that CDK7 plays a pivotal role in the processes of transcription and cell cycle regulation, and furthermore, it might be associated with HCC progression [14,33]. In this study, we used HCC data from TCGA and GEO to screen and identify eight prognostic genes related to CDK7 in HCC using bioinformatics methods with the aims of uncovering potential therapeutic targets and improving diagnostic accuracy for HCC. Our findings contribute to existing knowledge by elucidating molecular interactions that can be used to improve patient outcomes.

This study identified eight genes (*CHGA*, *SH2D5*, *ACTBP12*, *SOX2*, *AGR2*, *ISM2*, *KRT12*, and *BRDT*) as prognostic markers through differential expression analysis and other methods. A multigene prognostic model was constructed to investigate their combined predictive value for predicting the prognosis of patients with HCC. Each of these genes has been independently verified for its association with the biological characteristics of various cancers, and their impact in HCC is especially notable. *CHGA* belongs to the chromogranin/secretin family of neuroendocrine secreted proteins [34]. It is found in secretory vesicles of neurons and endocrine cells. *CHGA* is expressed in multiple cancer types, and it is associated with poor prognosis [34,35]. *SH2D5* encodes a protein induced by HBV that can bind to transketolase, activating STAT3 and its downstream signaling pathways, which in turn promotes the proliferation of HCC cells [36,37]. Furthermore, *SH2D5* expression in patients with HBV-related HCC is higher in liver tissues than in adjacent nontumorous tissues [37].

*SOX2*, a transcription factor in stem cells, is also a key regulator of the self-renewal and pluripotency abilities of cancer stem cells (CSCs) [38]. In several cancer types, *SOX2* expression is significantly associated with tumor progression and higher mortality rates [39,40,41]. The long-term recurrence and metastasis of HCC could be attributable to the regulatory role of CSCs. However, the regulatory and prognostic roles of *SOX2* in HCC are not well understood. Studies have demonstrated that *AGR2* is highly expressed in both HCC cell lines and patient samples. *AGR2* overexpression is correlated with the metastatic status of HCC cells, and siRNA-mediated inhibition of *AGR2* results in a significant reduction in the invasiveness of metastatic cells *in vitro* [42,43]. *AGR2* is believed to facilitate HCC invasion and distant metastasis by modulating the MAPK and caspase signaling pathways [44,45]. The aforementioned studies indicate that *CHGA*, *SH2D5*, *SOX2* and *AGR2* have certain roles in the development of HCC or other tumors. Conversely, research on *ACTBP12*, *ISM2*, *KRT12*, and *BRDT* in the context of HCC is relatively limited. Their incorporation into our prognostic model signifies a novel strategy aimed at enhancing the precision of outcome predictions for patients with HCC. This methodology surpasses the limitations inherent in models that depend solely on individual genes.

Additionally, a risk model was developed using these eight prognostic genes, classifying patients with HCC into high- and low-risk groups. The KM survival curve indicated that high-risk patients have a lower survival probability. Furthermore, the high-risk group exhibited elevated risk scores and reduced survival times. ROC curve analysis demonstrated that the AUC of the risk model exceeded 0.6 at 1, 3, and 5 years, indicating its moderate predictive power. Similarly, in the GSE14520 dataset, patients with HCC were classified into high- and low-risk groups. The results of KM survival curve, risk curve, and ROC curve analyses in this dataset were consistent with those observed in the TCGA dataset, further validating the reliable predictive capability of the risk model. In the future, the application of this risk model may provide patients with more accurate prognostic assessments, which could help inform personalized treatment planning. For high-risk patients, more aggressive treatment measures could be considered to slow disease progression, whereas more conservative strategies might be appropriate to avoid unnecessary overtreatment in low-risk patients.

Upon analyzing the frequencies of gene mutations in both the high- and low-risk groups, the results revealed that *TP53* was the most frequently mutated gene in the high-risk group (44%), whereas *CTNNB1* mutations were most common in the low-risk group (32%). Notably, *TP53* mutations have a substantial impact on the expression of immune checkpoint molecules across various cancers [46,47]. The wild-type p53 protein is crucial for regulating apoptosis and the cell cycle following DNA damage. When *TP53* is mutated, cells with damaged DNA might evade apoptosis and develop into cancer cells [48]. In HCC, *TP53* is correlated and factors such as serum alpha-fetoprotein levels, tumor staging, vascular invasion, tumor differentiation, and the Child–Pugh classification [49]. A recent investigation indicated that various immune responses are linked to the mutational status of *TP53*. *TP53* mutations or deletions enhance programmed cell death ligand 1 expression and diminish the infiltration of CD8^+^ T cells in HCC. HCC lacking *TP53* mutations exhibits a more robust immune response than HCC with *TP53* mutations [50,51]. Our study found a significantly high frequency of *TP53* mutations within the high-risk group, aiding in the identification of patients with HCC at greater risk for poor outcomes.

Beyond the prevalent driver mutations in *TP53* and *CTNNB1*, our mutation landscape analysis also revealed missense mutations in several genes comprising the prognostic signature, such as *CHGA*, *AGR2*, and *SH2D5* (Fig. 8c). Whereas the functional impact of these specific mutations in HCC requires experimental validation, their occurrence in genes previously linked to cancer progression [52,53,54] provides a plausible genomic dimension to our expression-based risk model. This suggests that genetic alterations, in addition to transcriptional dysregulation, of these signature genes might collectively underlie the aggressive clinical behavior identified in high-risk patients.

Gain-of-function (GOF) mutations in *CTNNB1* are among the most frequent genetic alterations observed in HCC. GOF *CTNNB1* mutations lead to the activation of β-catenin, which then promotes the expression of TBX3. TBX3 upregulation inhibits phospholipase D1 (PLD1). Decreased PLD1 expression activates LATS kinases, ultimately resulting in the suppression of YAP and TAZ. This molecular pathway could contribute to the preservation of a less aggressive phenotype in HCC [55,56]. Although our study found that *CTNNB1* was the most frequently mutated gene in the low-risk cohort, its potential role as a risk factor for HCC cannot be ignored. Monitoring and evaluating *CTNNB1* mutations require high attention in patients with HCC [57,58]. Subsequently, we integrated clinical characteristics with the prediction model and identified two independent prognostic factors (risk score and T stage) through multivariate Cox analysis. Significant survival differences were observed among the clinical characteristic subgroups of the high- and low-risk groups. Notably, the low-risk cohort exhibited a higher probability of survival in these subgroups. Therefore, our model holds guiding significance for predicting the survival of patients with different clinical characteristics.

Through *in vivo* and *in vitro* experiments, the findings suggested that *CDK7* inhibited HCC cell proliferation, migration, and invasion and suppressed tumor growth by regulating the phosphorylation of p65. *CDK7* overexpression promoted the malignant phenotype of HCC. NF-κB is a key regulator of inflammation and cell death, and it is involved in liver cell damage, liver fibrosis, and liver cancer. NF-κB is regarded as a potential target for the prevention and treatment of HCC [59,60]. Our initial bioinformatics analysis, including GSEA, indicated that high *CDK7* expression was associated with alterations in various fundamental pathways such as complement and coagulation cascades (Fig. 1c), suggesting its broad impact on the HCC cellular state. To explore a potential mechanistic axis, we focused on transcriptional regulation and found that CDK7 expression is associated with RelA/p65 phosphorylation, which correlates with HCC progression (Fig. 10). As one of the most important members of the NF-κB family, NF-κB-p65 (RelA) is one of the five subunits activating the NF-κB pathway to regulate downstream gene expression. Therefore, RelA appears to be a key molecule that promotes the occurrence and progression of HCC [61]. Our experiments indicated that changes in *CDK7* expression are associated with the phosphorylation status of RelA/p65, and this association is correlated with the malignant phenotypes of HCC. However, further studies are needed to clarify causality.

A major question arising from our findings is whether *CDK7* has been uncovered here, satisfaction biomarker and functional driver, is targeted in HCC. *CDK7* is a CAK that is involved in the activation of cell cycle CDKs (e.g., CDK1, CDK2). In addition, *CDK7*, a core component of the transcription factor TFIIH, phosphorylates RNA polymerase II to regulate gene transcription [15,62]. The dual capacity of *CDK7* to stimulate the cell cycle and promote the expression of pro-survival oncogenes has rendered it a druggable target. These processes utilize the same molecular pathway. Thus, arrest of the CDK7 pathway disrupts both oncogenic signaling in cancer cells and cell cycle progression. Therefore, halting both processes is possible because of a phenomenon called transcriptional addiction [11,63]. In light of our findings that *CDK7* knockdown suppresses HCC cell proliferation, migration, and invasion *in vitro* and reduces tumor growth *in vivo* in HCC, CDK7 inhibition could represent a promising treatment strategy to counter HCC aggressiveness.

Previous studies have shown that CDK7 serves as an effective target for chemosensitization. PTCL is sensitive to transcription-targeted drugs, particularly to THZ1, a covalent inhibitor of CDK7 [64]. This induction was caused at least in part by suppression of the STAT3–MCL1–CHK1 axis, leading to increases in apoptosis and DNA damage. This finding is directly relevant to the current study, as we observed a significant positive correlation between *CDK7* and *SH2D5*, which encodes an HBV-induced protein known to activate STAT3 signaling [36,37]. This suggests a potential mechanism whereby CDK7 can antagonize STAT3, which was also similarly affected by CDK7 in our functional analyses. The convergence of STAT3 signaling across different cancer types intensifies CDK7 inhibition, which might represent a widely applicable strategy to disrupt this key oncogenic pathway.

Recent research has highlighted CDK7 as a promising therapeutic target for HCC. For instance, studies have found that *CDK7* is overexpressed in HCC, and its inhibition by THZ1 decreases tumor growth by inhibiting key oncogenic transcriptional programs [65,66]. Our study revealed that *CDK7* expression was significantly elevated, and this increased expression portended poor prognoses. Several small-molecule CDK7 inhibitors are being investigated from a pharmacological perspective. The clinical translation of THZ1 is viable through the use of AT7519 and CT7001 (samuraciclib), which are inhibitors with enhanced pharmaceutical properties [67,68]. Notably, a recent study [69] demonstrated that AT7519 functions as a RORγt agonist, reducing PD-1 and CTLA-4 expression and enhancing anti–PD-1 efficacy in HCC, revealing an additional immunological mechanism. This aligns with our exploratory findings on CDK7-associated immune infiltration (e.g., differential M1 macrophages), suggesting that CDK7 inhibitors might exert synergistic antitumor effects via immune microenvironment remodeling. Further, CT7001 has entered into clinical trials for advanced solid tumors [70]. RelA/p65 phosphorylation is involved in our model, and exploring synergistic combinations of CDK7 inhibitors and agents that target the NF-κB pathway, such as proteasome inhibitors or IKK inhibitors, could be a promising avenue for future research [14].

In the near future, our eight-gene signature is expected to be utilized as a prognostic tool and in patient selection for future clinical trials of CDK7 inhibitors. Individuals classified as high-risk because of the activation of CDK7 and downstream pathways might potentially benefit from CDK7 inhibitor therapy, although patient selection requires validation in prospective clinical trials. In addition, through computer simulation screening of chemical and biological databases (such as the Cancer Drug Sensitivity Genomics Database and Cancer Treatment Response Portal), more small-molecule compounds targeting CDK7 or its downstream effector factors (e.g., SH2D5, MCL1) could be discovered, and these compounds have exhibited high efficacy in HCC models. Through the construction of prognostic models with clear mechanisms and pharmacological principles, this comprehensive approach is expected to exceed static risk prediction and reveal new dynamic models that can guide the management of HCC.

## 5 Conclusions

In conclusion, bioinformatics analysis combined with experimental verification suggested that CDK7 is a prognostic factor for HCC. We constructed a risk model based on an eight-gene prognostic signature (*CHGA*, *SH2D5*, *ACTBP12*, *SOX2*, *AGR2*, *ISM2*, *KRT12*, and *BRDT*) that stratifies patients into distinct risk cohorts with significant survival differences and validated its categorical predictive performance in an independent cohort. *TP53* mutations, which give rise to immunosuppressive microenvironments, were frequent in the high-risk group. Functional experiments demonstrated that CDK7 is associated with the phosphorylation of RelA (p65), and this association might be involved in HCC proliferation, migration, and tumor growth, making it a potential therapeutic target, but further studies are needed to clarify causality. Our expanded discussion, integrating our own prior work and the latest literature, provides a pharmacological rationale for targeting CDK7 in HCC. This discussion highlights the potential for repurposing existing CDK7 inhibitors and exploring rational drug combinations, thereby bridging the gap between prognostic biomarker discovery and the development of novel therapeutic strategies.

However, this study has several limitations. First, the analysis of public cohorts (TCGA, GSE14520) was retrospective, which might have introduced selection bias. Second, the risk model was only externally validated in the GSE14520 cohort, which has a limited sample size. Third, the association between CDK7 and RelA/p65 phosphorylation is correlative, and causality has not been established by rescue assays or nuclear translocation analysis. Fourth, only murine cell lines (HEPA1-6, Hepa1c1c7) were used in the functional experiments, and species differences can affect the extrapolation of these results to human HCC. Future prospective studies, larger validation cohorts, refined mechanistic experiments, and the use of human cell lines are needed to further verify the conclusions. Overall, our results provide a novel prognostic multigene platform for HCC and mechanistically suggest that CDK7 inhibition could have therapeutic benefits.

## Data Availability

The data that support the findings of this study are available from the Corresponding Author, Siyuan Zeng, upon reasonable request.

## Supplementary Materials

The supplementary material is available online at https://www.techscience.com/doi/10.32604/or.2026.081711/s1.

## Abbreviations

The following abbreviations are used in this manuscript:

CDK7	Cyclin-dependent kinase 7
HCC	Hepatocellular Carcinoma
KM	Kaplan-Meier
DEG	Differentially expressed genes
HBV	Hepatitis B virus
HCV	Hepatitis C virus
NAFLD	Nonalcoholic fatty liver disease
TCGA	The Cancer Genome Atlas
GSEA	Gene set enrichment analysis
ROC	Receiver operating characteristic
KEGG	Kyoto Encyclopedia of Genes and Genomes
