# Supplementary figures and images for: Integrative Analysis Identified an Eight-Gene Risk Signature Linked to CDK7 and Explored Its Association with HCC Progression via RelA Phosphorylation
Source: Oncol Res. 2026 Jul 16;34(8):18. doi: 10.32604/or.2026.081711 (PMC13397321; doi:10.32604/or.2026.081711)

HEPA1-6 si-CDK7

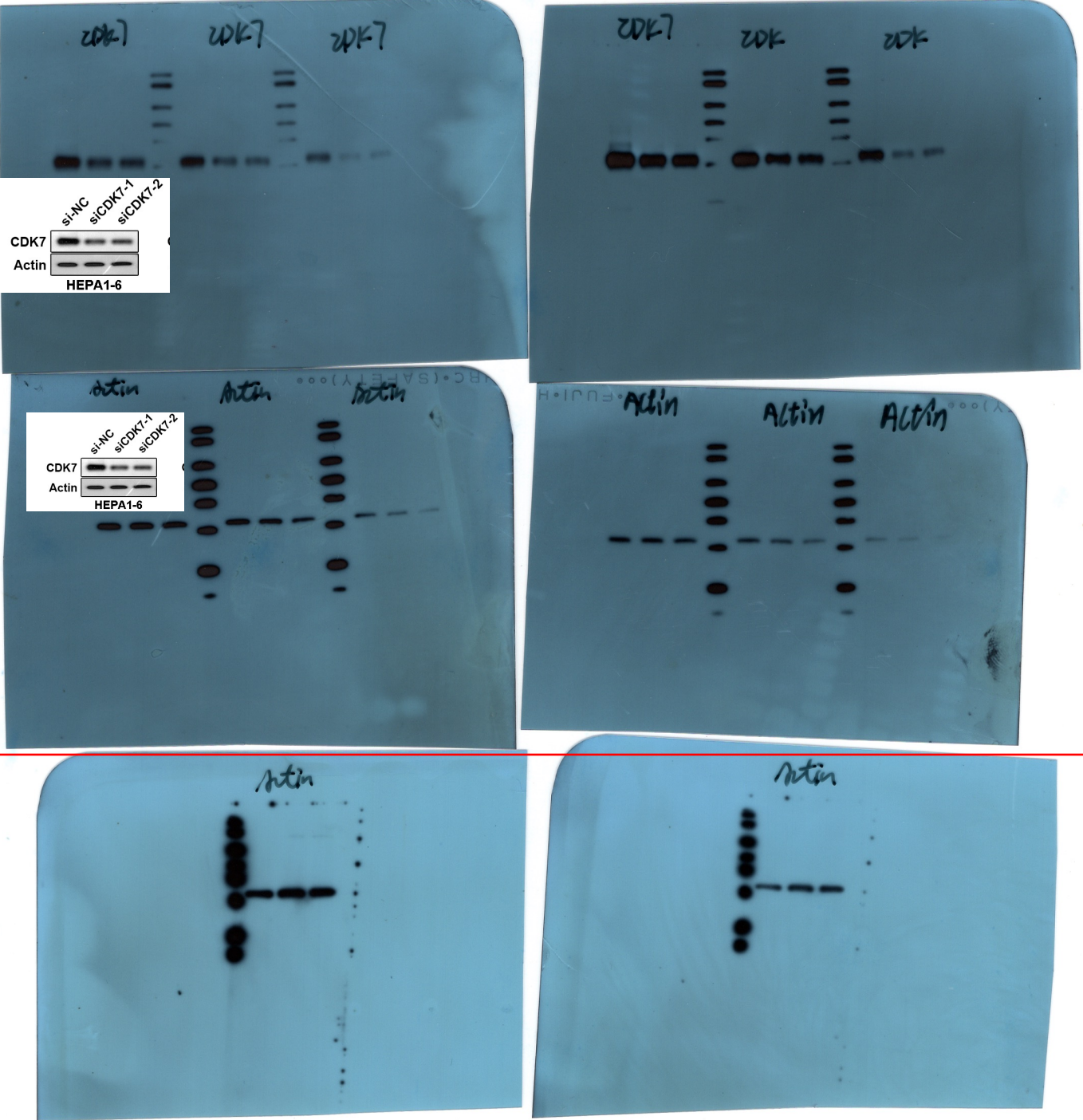

Hepa1c1c7 si-CDK7

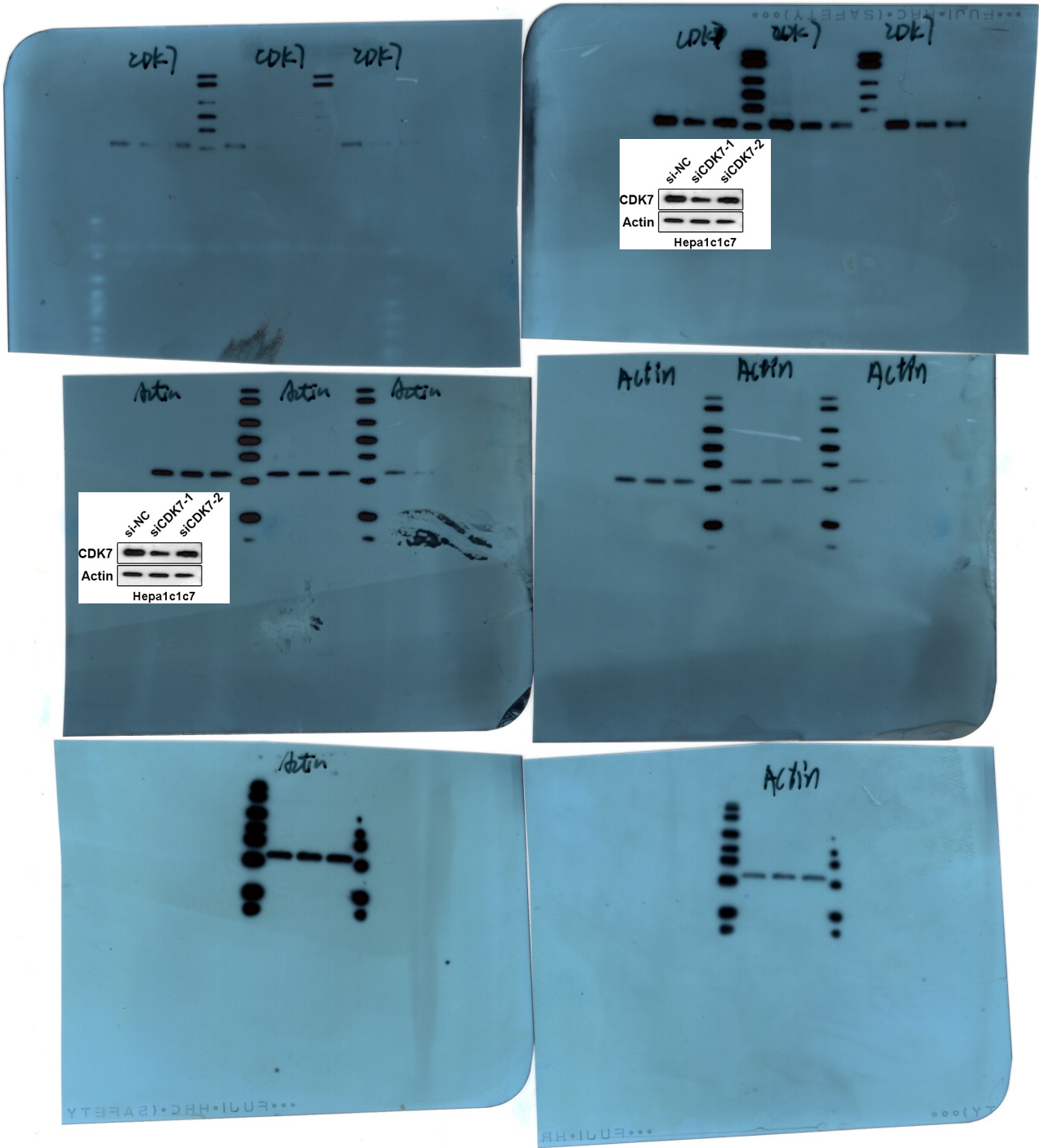

HEPA1-6 CDK7-OE

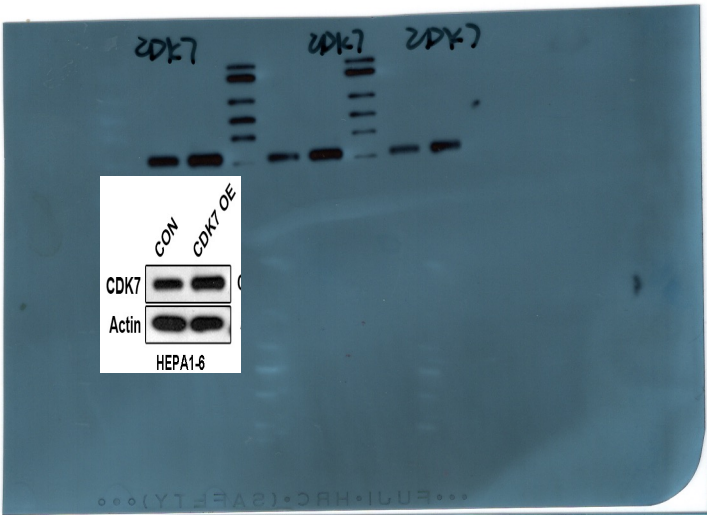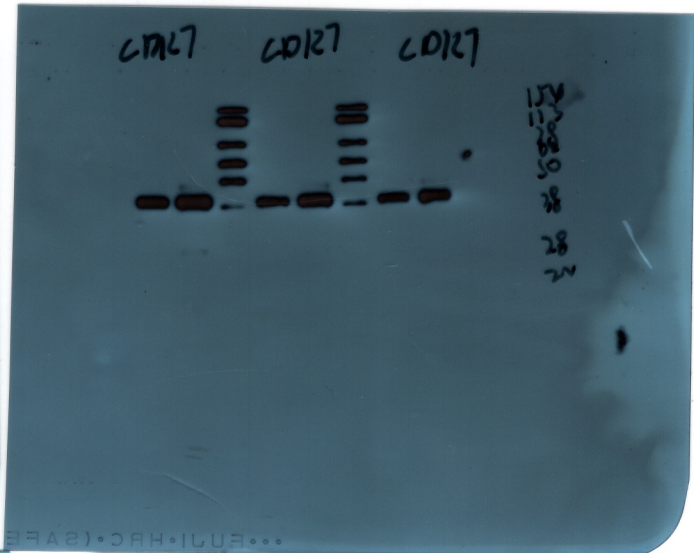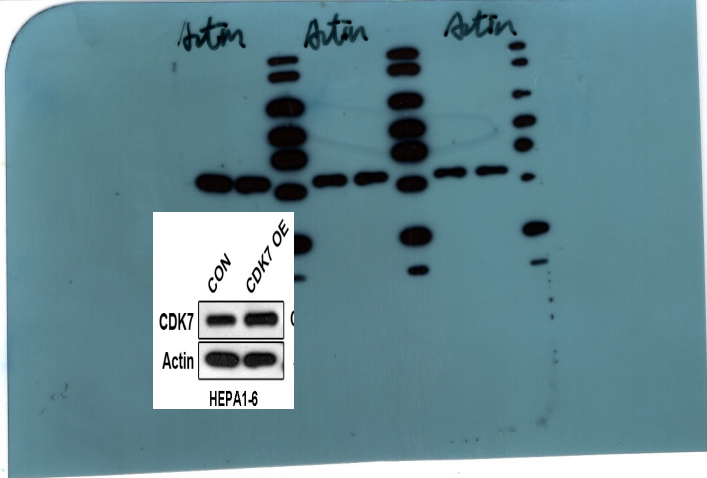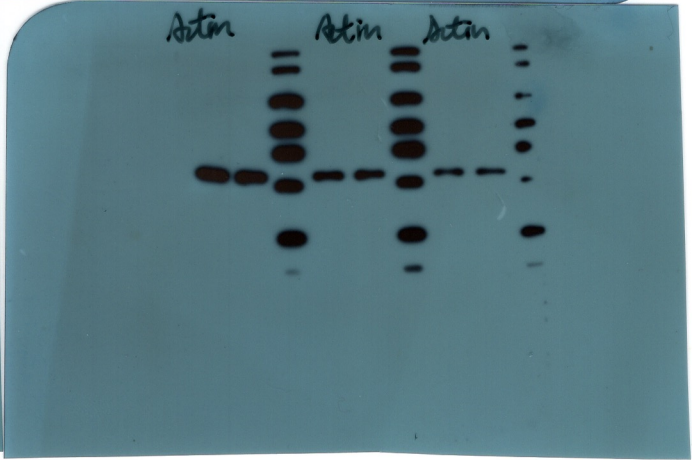

Hepa1c1c7 CDK7-OE

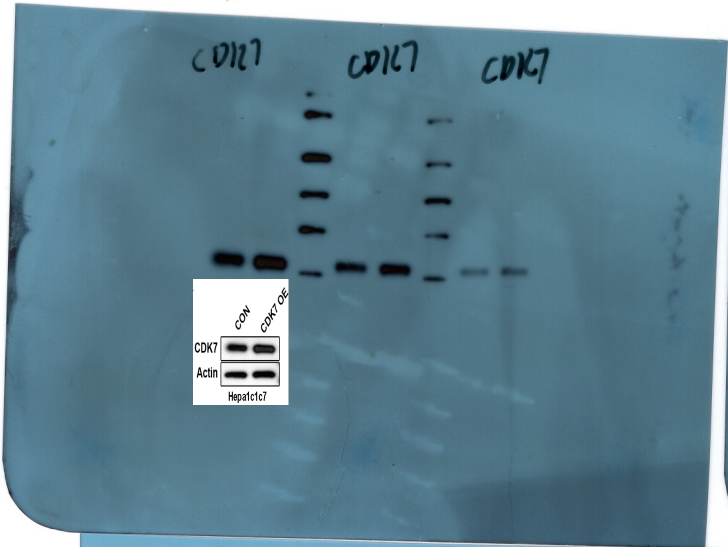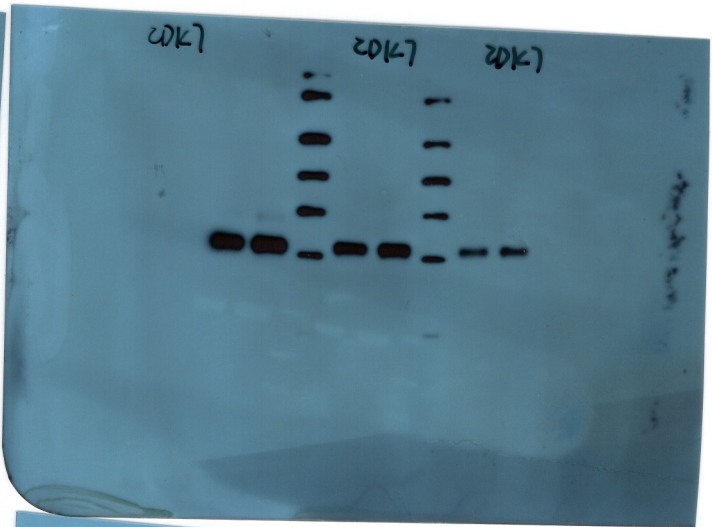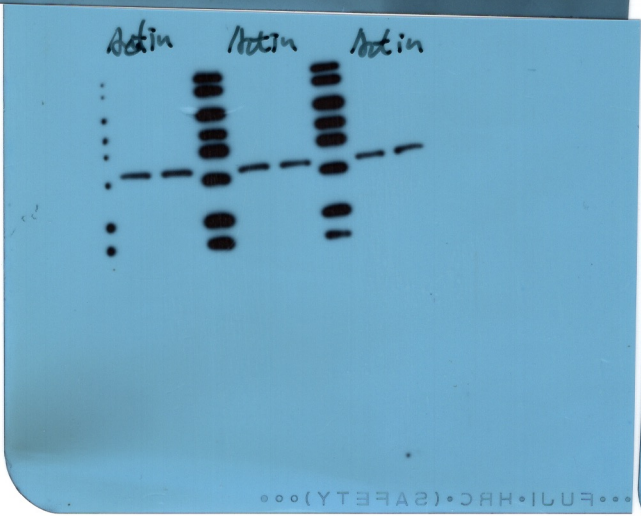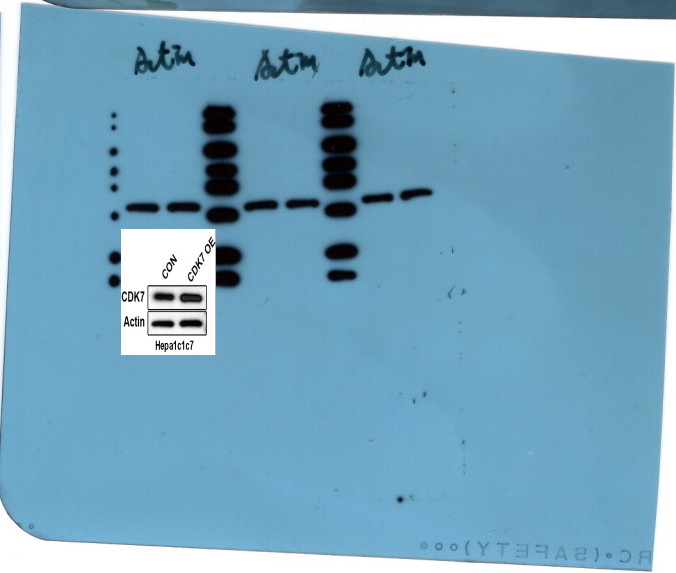

si-CDK7. p65. p-p65

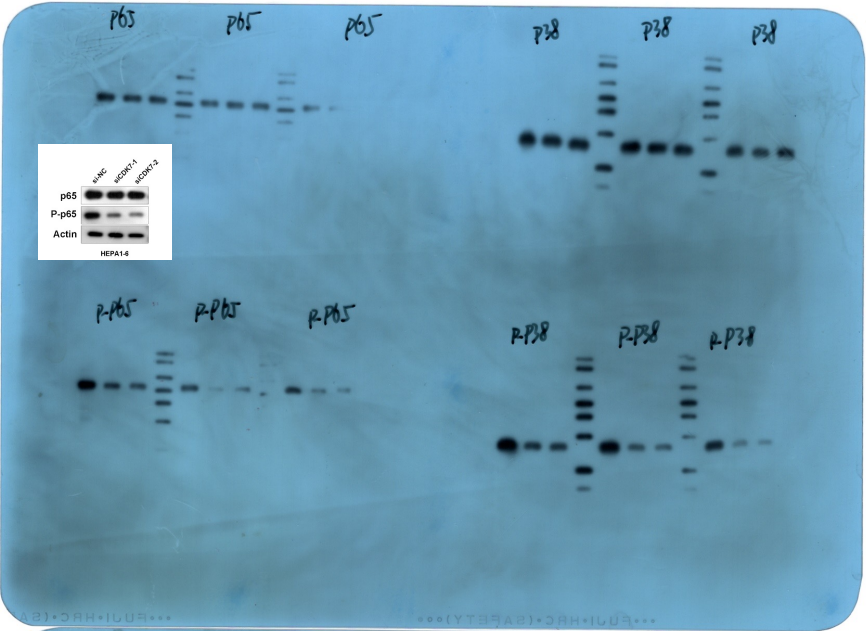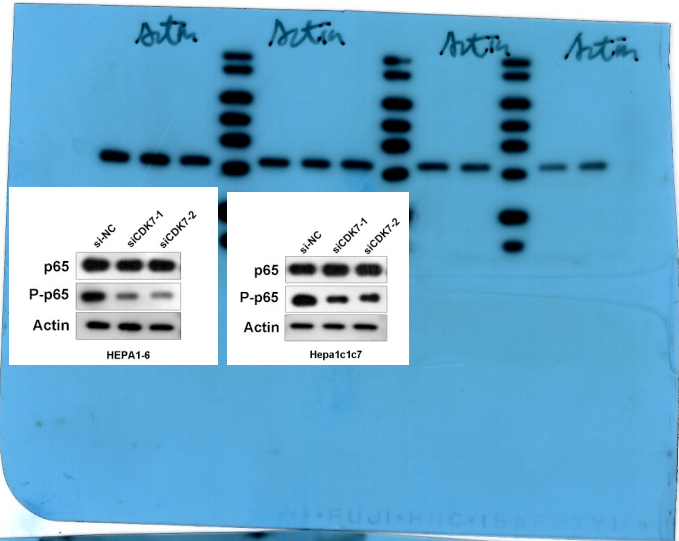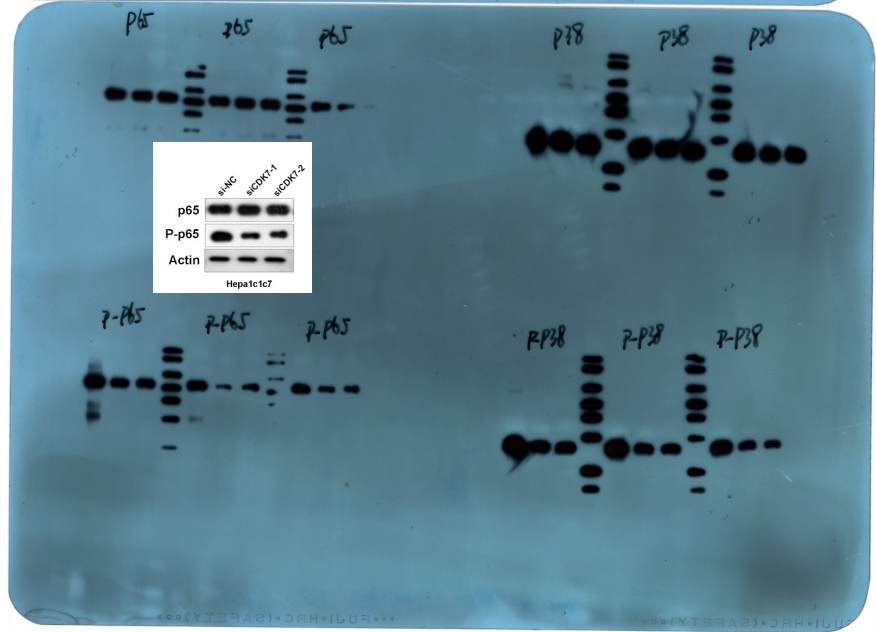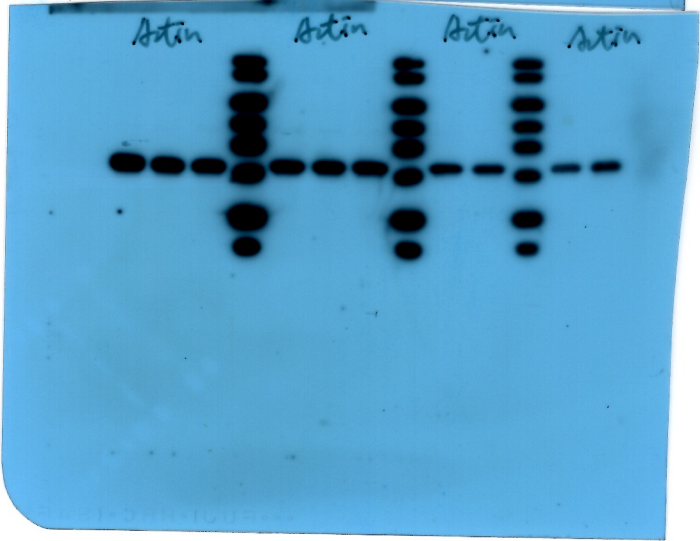

CDK7-OE. p65. p-p65

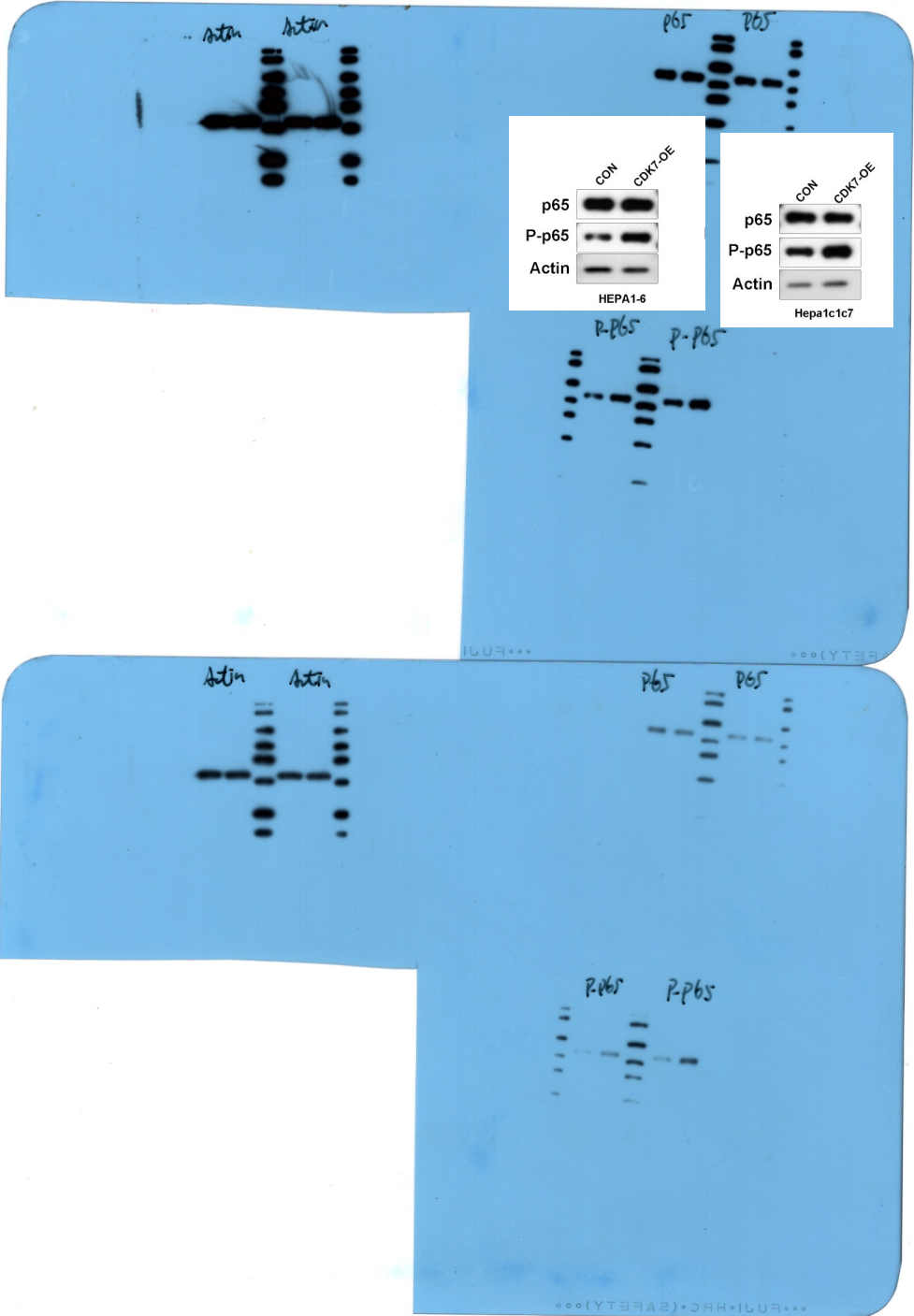

Supplement: Supplementary file 1 [file OncolRes-34-81711-s001.zip › Supplementary_File_S1.pdf]
